# Csu pili dependent biofilm formation and virulence of *Acinetobacter baumannii*

**DOI:** 10.1038/s41522-023-00465-6

**Published:** 2023-12-14

**Authors:** Irfan Ahmad, Aftab Nadeem, Fizza Mushtaq, Nikola Zlatkov, Muhammad Shahzad, Anton V. Zavialov, Sun Nyunt Wai, Bernt Eric Uhlin

**Affiliations:** 1 Department of Molecular Biology and Umeå Centre for Microbial Research (UCMR), Umeå University, SE-90187 Umeå, Sweden; 2 Institute of Biomedical and Allied Health Sciences, University of Health Sciences, Lahore, Pakistan; 3 Department of Pharmacology, University of Health Sciences, Lahore, Pakistan; 4 Department of Biochemistry, University of Turku, Tykistökatu 6A, 20520 Turku, Finland; 5 The Laboratory for Molecular Infection Medicine Sweden (MIMS), Umeå University, SE-90187 Umeå, Sweden

**Keywords:** Bacteriology, Biofilms

## Abstract

*Acinetobacter baumannii* has emerged as one of the most common extensive drug-resistant nosocomial bacterial pathogens. Not only can the bacteria survive in hospital settings for long periods, but they are also able to resist adverse conditions. However, underlying regulatory mechanisms that allow *A. baumannii* to cope with these conditions and mediate its virulence are poorly understood. Here, we show that bi-stable expression of the Csu pili, along with the production of poly-N-acetyl glucosamine, regulates the formation of Mountain-like biofilm-patches on glass surfaces to protect bacteria from the bactericidal effect of colistin. Csu pilus assembly is found to be an essential component of mature biofilms formed on glass surfaces and of pellicles. By using several microscopic techniques, we show that clinical isolates of *A. baumannii* carrying abundant Csu pili mediate adherence to epithelial cells. In addition, Csu pili suppressed surface-associated motility but enhanced colonization of bacteria into the lungs, spleen, and liver in a mouse model of systemic infection. The screening of c-di-GMP metabolizing protein mutants of *A. baumannii* 17978 for the capability to adhere to epithelial cells led us to identify GGDEF/EAL protein AIS_2337, here denoted PdeB, as a major regulator of Csu pili-mediated virulence and biofilm formation. Moreover, PdeB was found to be involved in the type IV pili-regulated robustness of surface-associated motility. Our findings suggest that the Csu pilus is not only a functional component of mature *A. baumannii* biofilms but also a major virulence factor promoting the initiation of disease progression by mediating bacterial adherence to epithelial cells.

## Introduction

*Acinetobacter baumannii* has emerged as one of the most common nosocomial pathogens in many developing countries. It is found to be associated with several kinds of human infections, such as meningitis, ventilator-associated pneumonia, septicaemia, skin infections, and urinary tract infections^1,2^. The genus *Acinetobacter* consists of at least 23 species^3^ but it is the outbreaks specifically linked to *A. baumannii* that have imposed a major burden on the health care system in recent years^4,5^. Furthermore, *A. baumannii* has been identified as one of the most prevalent causes of secondary infections in COVID-19 patients^6–10^. The pathogenic potential of *A. baumannii* is determined by several properties, including presumed virulence factors, e.g. outer membrane protein A (OmpA)^11^, K1 capsular polysaccharide, lipopolysaccharides, and extracellular appendages as pili^2,12^. Furthermore, plasmids harbouring genes for organic peroxide resistance have been associated with the virulence potential of *A. baumannii*^13,14^. Its ability to colonize and survive on a wide range of biotic and abiotic surfaces, as well as its increased biofilm-forming capacity, play a pivotal role in making it one of the most persistent and antimicrobial-resistant pathogens^5,11,15^.

Two major types of adhesive pili have been described in the case of *A. baumannii:* the Csu pili found to mediate attachment and biofilm formation on abiotic surfaces^16^, and the type IV pili involved in twitching motility^17^. The potential roles of these pili in adherence to human epithelial cells and tissues during infection have been unclear^18,19^. The type IV pili of *A. baumannii* are shorter (5–140 nm) than the Csu pili (140–1000 nm) and are shown to be essential for both twitching motility and natural competence^20^. Adherence of *A. baumannii* to epithelial cells is promoted by the type IV pili^21^. The major subunit, PilA, from the hypervirulent strain *A. baumannii* AB5075 and the one from the multidrug-resistant strain ACICU restores the adherence defect of the *pilA* deletion mutant in *A. baumannii* M2^22^. However, protein sequence diversity exists among the major subunit (PilA) of type IV pili among different species of *Acinetobacter* and therefore, further investigations are required to determine its precise role in the adherence phenotype.

The Csu pili are assembled via the chaperon-usher pathway (CUP) and belong to the Archaic CUP pili that constitute the largest family of CUP pili in bacteria^23^. The assembly apparatus of Csu pili is encoded by an operon that consists of six genes: *csuA/B, csuA, csuB, csuC, csuD*, and *csuE*^24,25^. The archaic systems have a far wider phylogenetic distribution and are associated with a broader range of diseases than their classical equivalents^25^. In the Csu pilus polymer, CsuA/B subunits are linked by donor strand complementation (DSC)^23,26,27^, with the N-terminal sequence of one subunit inserted into the hydrophobic cleft of a neighbouring subunit^24^. Recent structural and biomechanical studies of the Csu pili indicate that they are formed as super-elastic zigzag springs^28^. The CsuE subunit is located at the tip of the pilus and confers adhesive properties to the pilus^23,24^. Via Csu pili, *A baumannii* can colonize the surfaces of medical euipment such as gloves^23^.

*A. baumannii* 17978 is shown to form mushroom-shaped biofilm structures when grown in a roller biofilm bioreactor^29^. The Csu pili promoted the formation of these mushroom-shaped structures in a cell density-dependent manner, regulated by the *luxR1*-type quorum sensing molecule Acyl homoserine lactone (AHL) synthase AbaI. The *abaI* gene is co-transcribed with the *abaM* gene coding for the AHL suppressor AbaM under the control of the transcriptional regulator AbaR^30^. The enhanced expression of AHLs in an *abaM::T26* mutant promoted biofilm formation on abiotic surfaces and suppressed *A. baumannii* virulence as monitored with the *Galleria mellonella* larvae infection model. Although AbaI and AbaM opposingly regulate the production of AHL, expression of the *csu* operon is enhanced in both mutants^30^. However, whether the Csu pili are involved in the attachment of bacteria to biotic surfaces and epithelial cells and thereby contribute to bacterial virulence has been unclear. In this regard, some clinical isolates of *A. baumannii* and *A. pittii* were shown to be incapable of adhering to epithelial cells and to induce a cytotoxic effect^31^. In contrast, the *A. baumannii* 19606 strain, which abundantly produces Csu pili, was found to adhere efficiently to epithelial cells^18^. On the other hand, a *csuE* mutant of *A. baumannii* 19606 did not show any defect in the adherence of lung epithelial cells^18^. Instead, due to some unidentified feedback regulation, the number of epithelial cells harbouring adhered bacteria was increased upon infection with the *ΔcsuE* mutant when compared to the parental strain19606^18^. Moreover, the apparent lack of expression of Csu pili by variants of *A. baumannii* 17978 enhanced the adherence of bacteria to epithelial cells, whereas the expression of *A. baumannii* Csu pilus assembly in *E. coli* JM109 facilitated the adherence of bacteria to A549 lung epithelial cells^32^. Hence, we considered that a detailed microscopic investigation involving mutants of genes encoding the major CsuA/B subunits would be required to clarify the role of the Csu pili in the adherence of *A. baumannii* in experimental settings where such pili are expressed. In addition, it has been shown recently that the subcellular localization of type IV pili controls the pattern of multicellular architecture in the development of complex bacterial communities^33^. However, little is known about the regulation and expression of Csu pili or their spatial involvement during mature biofilm formation.

Here, to evaluate their role (and spatial involvement) in biofilm formation, we present a comparative functional and regulatory analysis of the Csu and type IV pili under a variety of experimental conditions, aided by both in vitro and in vivo models. We identified the localization of Csu pili within mature biofilms formed on a glass surface and demonstrated their role in mediating adaptive drug resistance to the last line antibiotic colistin in the hyper virulent strain *A. baumannii* AB5075. We found that the Csu pili mediate the adherence of *A. baumannii* to epithelial cells and that the expression of Csu pili is associated with increased biofilm structural complexity, enhanced tolerance to colistin and virulence in vivo and decreased motility. The data suggest that the transition in the expression of two kinds of pili is regulated by the GGDEF/EAL protein AIS_2337, here denoted PdeB.

## Results

### Bi-stable expression of Csu pili and production of poly-N-acetyl glucosamine contribute to formation of dense patches of mature biofilm on glass surfaces

**Fig. 1:**
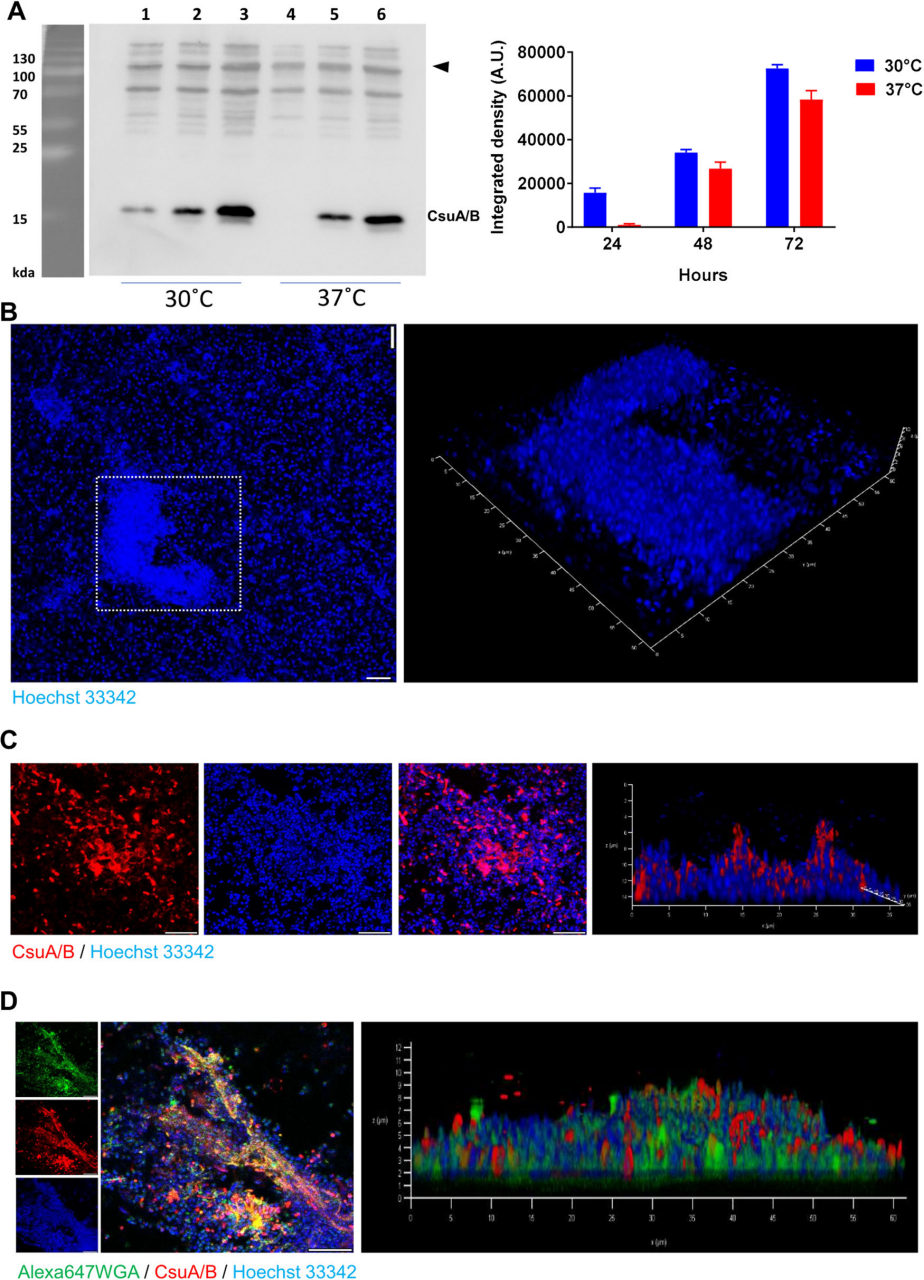
The expression of Csu pili and poly N-acetyl glucosamine in mountain-like biofilm patches formed by *A. baumannii* on glass surface. **A** Left panel: Representative Western blot of three independent experiments to show CsuA/B expression in biofilms formed by *A. baumannii* 17978 in glass tubes at different intervals of time. 1–24 h, 2–48 h, 3–72 h, 4–24 h, 5–48 h, 6–72 h. The arrowhead indicates equal distribution of proteins through a nonspecific band used as a loading control. Right panel: Quantification of the blots using image J software. Bars graph indicate mean +/– standard deviation (SD). **B** A confocal laser microscopic image of Hoechst 33342-labelled biofilm formed by *A. baumannii* 17978 on the surface of an 18-well chamber glass slide after 72 h. In the left image, a typical patch of biofilm is marked by the square of dotted white lines. The two size bars correspond to 10 µm. The image on the right shows a 3-D stake image of the spotted patch. **C** A single and multiple staked 2-D view of biofilm patches labelled with Hoechst 33342 and CsuA/B specific antibodies. The CsuA/B positive matrix is shown in red. The size bars correspond to 10 µm. **D** A single and multiple staked 2-D view of biofilm patches labelled with Hoechst 33342, CsuA/B specific antibody and WGA. The WGA-bound fluorescent matrix representing poly B-acetyl glucosamine is shown in green. The size bars in the images correspond to 10 µm.

*A. baumannii* has the ability to form biofilms on a wide range of surfaces, presumably making the biofilm phenotype an important virulence factor during infections^2^. To assess the importance of the Csu pili in biofilm formation, we investigated the expression of the CsuA/B protein at different time intervals, either at body temperature (37 °C) or at ambient temperature (30 °C). In accordance with their quorum sensing regulation^29,30^, we observed by western blot analysis that the expression of CsuA/B was cell density-dependent at both temperatures (Fig. 1A). Importantly, the expression of CsuA/B appeared to be at its maximum after 72 h of incubation in our experimental settings. We therefore monitored biofilm formation of *A. baumannii* 17978 for up to 72 h on 18-well chamber glass slides. The mature biofilm was visualized using confocal laser microscopy. The 3D visualization of biofilm structures revealed that the *A. baumannii* 17978 bacteria formed biofilm on glass surfaces in the form of “Mountain-like” patches (Fig. 1B). Such type of mature biofilm was not observed in a microfluidic flow cell chamber and it was clearly different from the macroscopically visible mushroom-shaped biofilm structures formed in rolling biofilm bioreactors^29^. Furthermore, in our experimental setting of 18-well chamber glass slides, the mushroom-shaped structures were not formed. To determine the localization of the components of the Csu pili in the biofilm, the mature biofilm was subjected to immunostaining with CsuA/B antiserum. By confocal laser microscopy, we observed that the detection of CsuA/B was limited to the mountain-like biofilm patches. The 3D visualization further indicated that the Csu pili were abundantly present on bacteria located particularly at the top of the mountain-like patches (Fig. 1C). The cells located at the base of those patches, near or on the glass surface, lacked detectable Csu pili (Fig. 1C). So far, our data support the previous findings that the Csu pili adherence occurs preferentially on hydrophobic surfaces rather than hydrophilic surfaces such as glass^23^. The observed formation of a mature biofilm on the glass surface that involved the Csu pili prompted us to consider if these pili might contribute to biofilm maturation via interaction with a hydrophobic extracellular matrix component. Poly N-acetyl glucosamine (PNAG) is a known extracellular matrix component of the hydrophobic biofilm of *A. baumannii*, and the lectin wheat germ agglutinin (WGA) binds selectively to PNAG^34^. WGA staining of biofilm patches revealed that PNAG indeed was abundantly present in the biofilm patches (Fig. 1D and Supplementary Fig. 1A). To address if CsuA/B and PNAG co-localize, WGA-positive biofilm was immunostained with CsuA/B antiserum and visualized by confocal laser microscopy. We observed that CsuA/B and PNAG co-localized in the subpopulation of bacteria that took part in the formation of the patches (Fig. 1D and Supplementary Fig. 1B). These findings led us to the conclusion that CsuA/B, the main component of the Csu pili, and the matrix component PNAG contribute to a hydrophobic mature biofilm matrix on the glass surface as mountain-like patches.

### Csu pili and type IV pili subunit PilA are inversely involved in the formation of mountain-like biofilm patches by *A. baumannii*

**Fig. 2:**
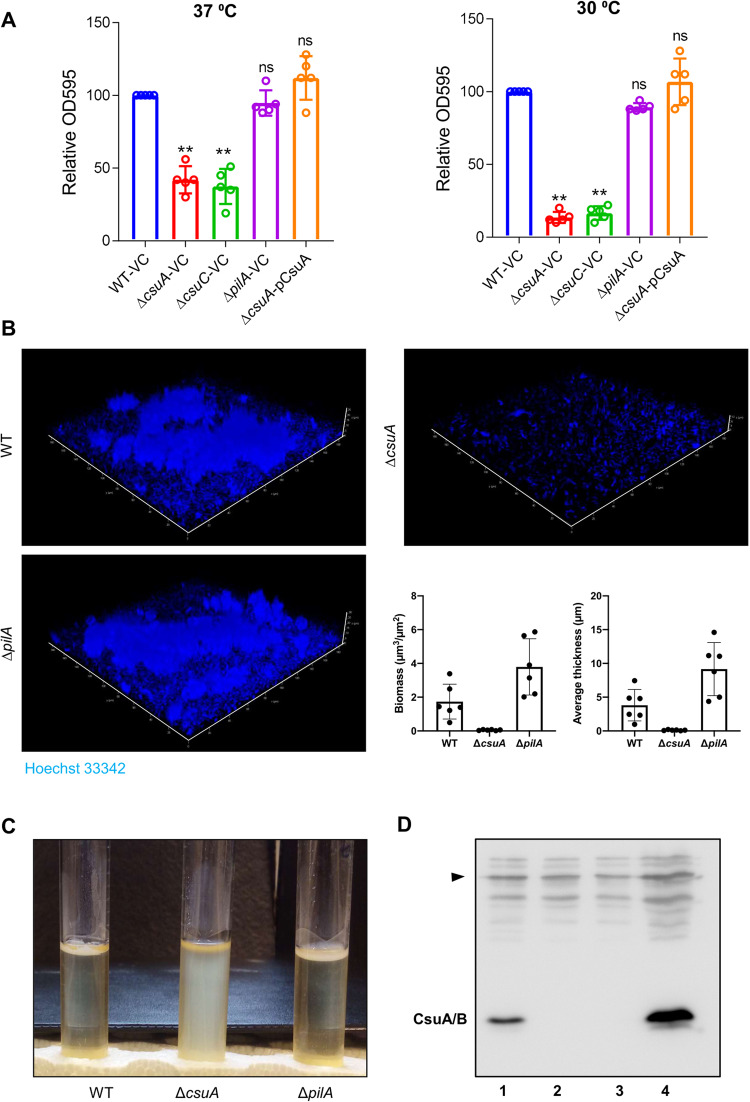
Csu pili, not type IV pili, are critical in the formation of biofilm patches. **A** Biofilm formation by *A. baumannii* 17978 wild-type (WT) and mutant strains was measured as the optical density of the crystal violet stained matrix of biofilm grown in glass tubes at 37 °C and 30 °C. Error bars show mean ± SD. Statistical significance is indicated by *p* value that was measured by non-parametric two tailed *t* test. Two asterisks ** indicate *p* ≤ 0.01 and “ns” indicates non-significant difference. **B** Three-dimensional confocal laser microscopic images of biofilm patches formed on glass in an 18-well camber by the wild-type strain and its *csuA* and *pilA* mutants. The patch formation was not observed in biofilms of the *csuA* mutant. The size bars in the images correspond to 10 µm. Histograms in the lower right panel indicate quantification of biomass and average thickness of the biofilm. Data are representative of two independent experiments; bar graphs show mean ± SD. Data points represent the quantification of data from six random fields of view. **C** Representative images of bacterial cultures grown statically in glass tubes as pellicles formed by *A. baumannii* wild-type and mutant strains at 30 °C after 72 h. **D** A representative Western blot image of three independent experiments shows the expression of the CsuA/B in *A. baumannii* 17978 wild type strain cultivated after the growth at different growth conditions. 1- LB broth after 48 h at 37 °C. 2- LB plates after 48 h at 37 °C, 3- LB agar plate lacking NaCl, 4- pellicle formed within LB without salt broth. The arrowhead indicates an equal distribution of proteins through a nonspecific band used as a loading control.

After addressing the role of Csu pili in the formation of mature biofilm patches by *A. baumannii*, we were interested in investigating whether type IV pili also might play a role. Therefore, mutant strains of *A. baumannii* 17978 lacking components of Csu or type IV pili were constructed by deleting the genes coding for CsuA, CsuC, and PilA. CsuA is an adaptor subunit, and its deletion abolishes the Csu pili assembly^23^. These mutations did not affect the growth of *A. baimannii* 17978 (Supplementary Fig. 1C). Then the ability of these mutants to form biofilm inside glass tubes was examined (Fig. 2A). After 72 h of incubation, biofilm formation by the *ΔcsuA* and *ΔcsuC* mutants was significantly lower than that of the wild type. The deletion of *csuA* reduced biofilm formation by *A. baumannii* 17978 in glass tubes at 30 °C by 84.4 (+/– 3,8) % and at 37 °C by 58.1(+/–, 9.2) % (Fig. 2A), whereas the deletion of *pilA* did not alter the level of biofilm formation in glass tubes. The biofilm formed by these different mutants at the bottom of the wells in an 18-well glass chamber was visualized by confocal laser microscopy. The patch formation was not detected in the biofilm formed by the *ΔcsuA* mutant, while the *ΔpilA* mutant formed intact patches (Fig. 2B). The changes in biofilm architecture calculated from 3D volume were further confirmed by COMSTAT analysis. We observed a reduction in biomass and average thickness of the biofilm in the *ΔcsuA* mutant compared to wild type or *ΔpilA* mutant strains of *A. baumannii* (Fig. 2B, bottom right panel). These findings suggest that the Csu pili are essential for the production of biofilm patches on glass surfaces. Interestingly, the *ΔpilA* mutant strain formed a thick pellicle at the air-liquid interface, whereas the *ΔcsuA* mutant did not (Fig. 2C). Further western blotting and microscopic analysis of the *ΔpilA* mutant confirmed that intact Csu pili are present even in the absence of PilA (Supplementary Fig. 2A–C). Western blotting analysis and microscopic analyses of these pellicles revealed that the level of CsuA/B was increased in the pellicle formed by the *ΔpilA* mutant when compared with the wild-type strain (Fig. 2D, Supplementary Fig. 2A–D). Surprisingly, the expression of CsuA/B was not detected in samples from colonies grown on LA plates both in the presence and absence of salt, although it was observed at high levels in bacterial samples after growth in LB (Fig. 2D and Supplementary Fig. 1B). Altogether, these data suggest that biosynthesis of the Csu pili is tightly regulated with respect to growth conditions, e.g., growth on solid or liquid media, and these pili are essential for the formation of mature biofilm patches on a glass surface and for pellicle formation at the air-liquid interface.

### Clinical isolates of *A. baumannii* express Csu pili that contribute to protection against bactericidal activity of colistin through formation of biofilm patches

**Fig. 3:**
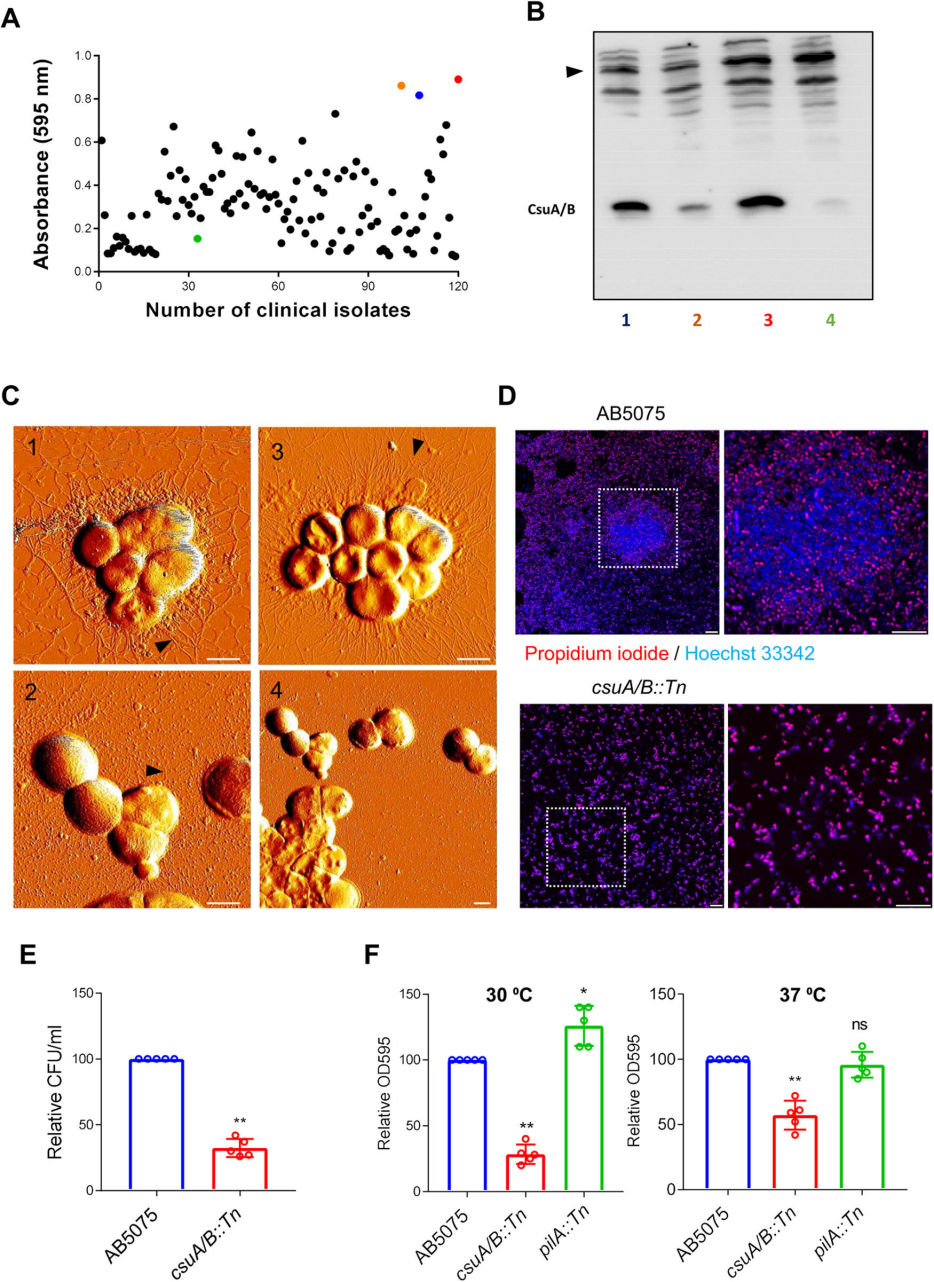
Clinical isolates of *A. baumannii* form CsuA/B-dependent biofilm patches that can protect bacteria from bactericidal effect of the anti-microbial drug colistin. **A** The distribution of intensity of biofilms produced by individual isolates in glass tubes upon the screening of 120 clinical isolates for biofilm production at 30 °C after 72 h. The optical densities shown here were calculated after subtracting the value for the *csuA* mutant of *A. baumannii* 17978, which was arbitrarily used as a baseline control. The coloured dots represent the isolates (blue, AB5075; orange, Ab-Pak-Pesh-37; red, Ab-Pak-Lah-14; green, Ab-pak-Pesh-22) selected for additional Western blot and AFM analyses. **B** Representative Western blot image of three independent experiments to show the CsuA/B expression in selected clinical isolates after 72 h 1- AB5075, 2- Ab-Pak-Pesh-37, 3- Ab-Pak-Lah-14, 4- Ab-Pak-Pesh-22. The arrowhead indicates an equal distribution of proteins through a nonspecific band used as loading control. **C** AFM images of clinical isolates grown in glass tubes for 72 h 1- AB5075, 2- Ab-Pak-Pesh-37, 3- Ab-Pak-Lah-14, 4- Ab-Pak-Pesh-22. Scale bars = 1 μm. **D** Confocal laser microscopic image of *A. baumannii* AB5075 biofilm patches labelled with Hoechst 33342 nuclear (blue) and Propidium Iodide (PI) staining after one hour of treatment with colistin at 5 µg/ml. The PI positive (dead) cells are shown in red. The size bars in the images correspond to 10 µm. **E** Bar chart diagram representing the number of viable cells (CFU/ml) in a 72-hour old biofilm culture of the wild-type and mutant strains upon colistin treatment. **F** Bar chart diagram comparing the biofilm formation of wild-type *A. baumannii* AB5075 to that of the AB5075 mutant strains in glass tubes. Bar graphs show mean ± SD. Statistical significance is indicated by *p* value that was measured by non-parametric two tailed *t* test. Two asterisks ** indicate *p* ≤ 0.01 and ns indicates non-significant difference.

To assess the relevance of Csu pili-mediated biofilm formation in clinical settings, we screened a collection of 120 *A. baumannii* clinical isolates for biofilm formation in glass tubes. We have described previously the molecular and clinical features of these isolates^35^. The optical density of the biofilm matrix upon staining with crystal violet was determined for each individual isolate (Fig. 3A). The results indicate that most of these isolates can form biofilm in glass tubes. The hyper biofilm forming strains Ab-pak-Lah-14 and Ab-pak-Pesh-37, the hypo biofilm-forming strain Ab-pak-Pesh-22 and the hyper-virulent reference strain AB5075 were further tested for the expression of Csu pili. We observed that the hyper biofilm-forming strains and the hyper-virulent strain had higher levels of CsuA/B expression (Fig. 3B). Consistent with the expression levels of CsuA/B, there appeared to be higher numbers of Csu pili on bacteria of those strains (Fig. 3C).

The hyper-virulent strain AB5075 was further investigated for the production and role of the Csu pili. Mutants of this strain with transposon insertions in the *csuA/B* and *pilA* loci were tested for their capacity to form pellicles at the air-liquid interface and biofilms in the glass tubes. Samples from the pellicles were immunostained and inspected by confocal laser microscopy for the expression of CsuA/B. The results revealed that, similar to the abovementioned observations with *A. baumannii* 17978 (Supplementary Fig. 2C). The levels of the CsuA/B pili were significantly higher in the *pilA* transposon mutant as compared to the wild type strain *A. baumannii* AB5075 (Supplementary Fig. 2E). Moreover, scanning electron microscopic (SEM) imaging of samples from pellicles confirmed the presence of long pili, i.e., Csu pili, in the *pilA* transposon mutant (Supplementary Fig. 2F). These findings suggest that the presence of PilA, i.e., the type IV pili, suppressed the expression of Csu pili also in *A. baumannii* AB5075. As observed in case of *A. baumannii* 17978, the expression of CsuA/B was primarily restricted to growth in LB and pellicle rather than during growth on agar plates in *A. baumannii* AB5075 (Supplementary Fig. 3F).

We further tested *A. baumannii* AB5075 wild-type and mutant strains for the formation of biofilm patches on glass surfaces. Consistent with the above-mentioned observations of strain17978, *A. baumannii* AB5075 grown on 18-well chamber glass slides showed clear formation of biofilm patches (Fig. 3D). Biofilms are known to contribute to antimicrobial resistance and hence we investigated the biological relevance of the observed *A. baumannii* biofilm patches, which formed upon colistin exposure for one hour. Interestingly, the bacteria inside patches remained alive after colistin treatment, whereas most bacteria outside of the patches lost viability, as indicated by the increase in propidium iodide (PI) uptake (Fig. 3D). In the case of the *csuA* mutant that did not form biofilm patches, over 80% of mutant bacteria were non-viable within 1 h of colistin treatment (Fig. 3E). Consistent with the observed decrease in biofilm formation by the *csuA* deletion mutant of *A. baumannii* 17978, the *csuA/B* transposon insertion mutant of *A. baumannii* AB5075 also exhibited deficient biofilm formation capacity on glass plates (Fig. 3F).

The impact of the Csu pili on biofilm formation was next assessed using a plastic growth chamber. For that purpose, the green fluorescent protein eGFP was expressed in *A. baumannii* 17978 and in the *ΔcsuA* and *ΔpilA* mutants. The bacterial strains were grown in the plastic growth chamber using LB as a growth medium. By confocal laser microscopy analysis, we confirmed that the colonization of the *csuA* mutant was less efficient in comparison to that of both wild-type and *ΔpilA* mutant strains (Supplementary Fig. 3). The treatment of biofilm bacteria with colistin for 2 h killed >70% of *ΔcsuA* mutant bacteria (Supplementary Fig. 3C). However, the immediate effect of colistin-mediated death on the wild-type bacteria was even less pronounced compared to the effect exerted on the *ΔpilA* mutant bacteria. We did not detect any effect of *csuA* or *pilA* mutations on the minimum inhibitory concentration of colistin and carbencillin (Supplementary Table 1). Altogether, these findings suggest that Csu pili contribute to the protection of *A. baumannii* from the bactericidal effect of colistin under biofilm-forming conditions.

### Csu pili play a crucial role in establishing adherence of *A. baumannii* to epithelial cells in vitro

**Fig. 4:**
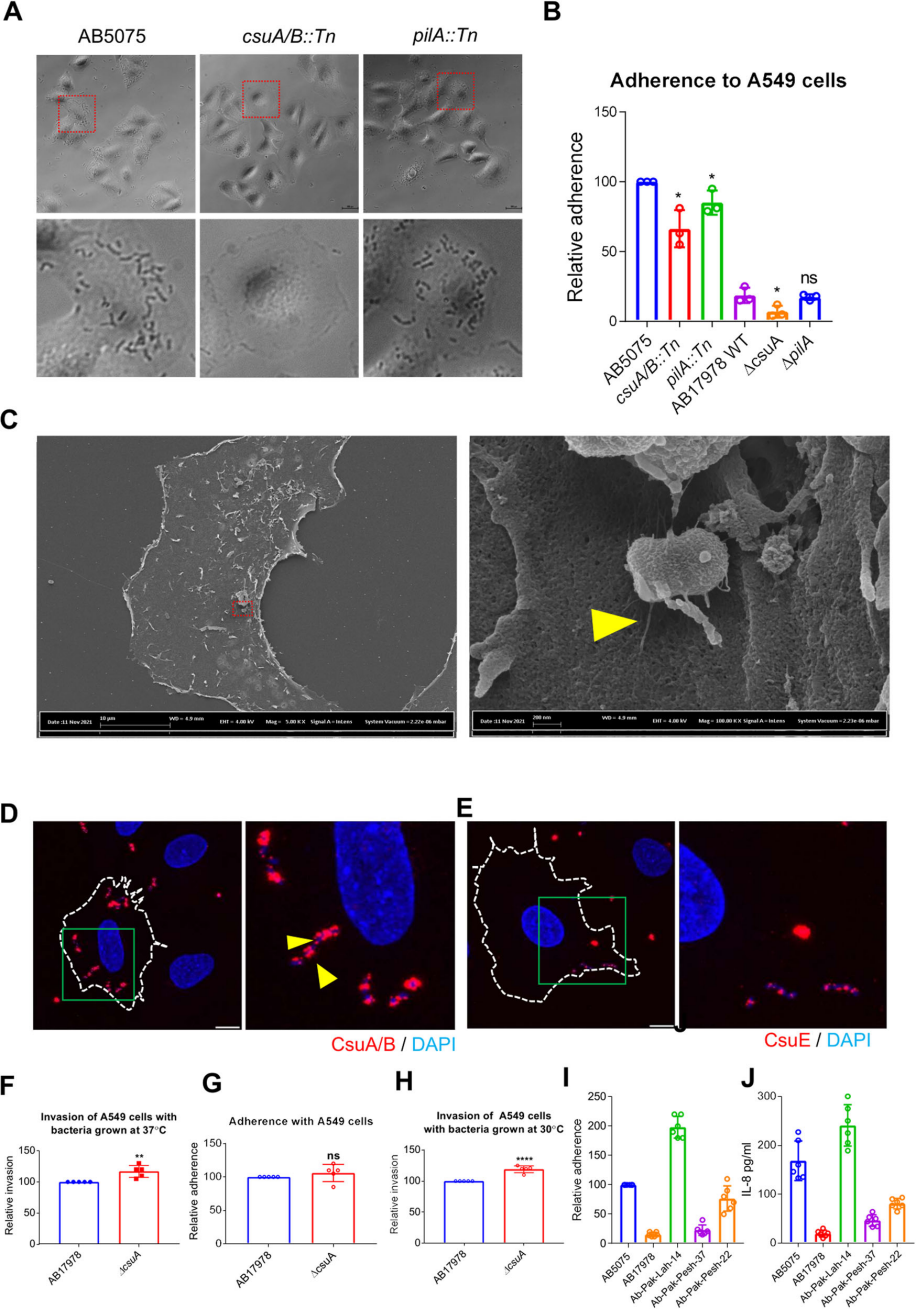
*A. baumannii* adheres to epithelial cells via Csu pili. **A** Left: Phase contrast microscopic images of *A. baumannii* AB5075 wild-type and mutant strains adhered to A549 epithelial cells after 4 h of infection with a MOI of 1:100. The lower row of images shows enlargements of the fields marked by dotted red lines in the upper row of images. **B** Bar chart diagram used to indicate the comparative analysis of the adherence capability of *A. baumannii* strains grown in LB broth over night at 30 °C with the epithelial cell line A549. **C** Left: A representative scanning electron microscopic image of *A. baumannii* AB5075 attached to A549 epithelial cells. Right: An enlargement of the area marked by dotted red lines. A presumed connection between bacteria and cells mediated by pili is highlighted by the yellow arrowhead. **D** Confocal laser microscopic images of *A. baumannii* AB5075 wild-type bacteria attached to epithelial cells labelled with primary antibodies raised against CsuA/B (**D**) and CsuE (**E**). The punctate staining spotted by arrow illustrates the expression of CsuA/B and CsuE on the surface of bacteria attached to epithelial cells. The size bars in the images correspond to 10 µm. **F** Bar chart diagram used to indicate the comparative analysis of the invasion capability of *A. baumannii* 17978 and *csuA* mutant strains grown in LB broth overnight at 30 °C to the epithelial cell line A549 (**G**). Bar chart diagram used to indicate the comparative analysis of the adherence capability of *A. baumannii* 17978 and *csuA* mutant strains grown in LB agar plates overnight at 37 °C to the epithelial cell line A549. **H** Bar chart diagram used to indicate the comparative analysis of the invasion capability of *A. baumannii* 17978 and *csuA* mutant strains grown in LB agar plates overnight at 30 °C into epithelial cell line A549. **I** Bar chart diagram used to indicate the comparative analysis of the adherence capability of *A. baumannii* strain AB5075 and other selected clinical isolates to the epithelial cell line SW480. **J** Induction of the cytokine IL-8 in SW480 cells upon infection with different *A. baumannni* isolates. Error bars show mean ± SD. Statistical significance is indicated by *p* value that was measured by non-parametric two tailed *t* test. A single asterisk * indicates *p* ≤ 0.05, two asterisks ** indicate *p* ≤ 0.01, four-asterisks **** indicate *p* ≤ 0.001 and “ns” indicates non-significant difference.

Besides the role in biofilm formation, bacterial pili are considered as virulence factors required for host cell adherence^36–39^. We therefore assessed the role of the Csu pili in *A. baumannii* adherence to epithelial cells in vitro. The wild-type *A. baumannii* strains AB5075 and AB17978 and its mutants were tested for their ability to adhere to the lung cancer epithelial cell line A549. The adherence capability of AB17978Δ*csuA* and AB5075-*csuA/B::Tn* mutants was significantly decreased when compared to their wild-type parental strains AB17978 and AB5075, whereas the decrease in adherence capability of the *pilA* mutants was not statistically significant (Fig. 4A, B). SEM imaging of *A. baumannii* AB5075 further revealed bacteria attached to epithelial cells with the help of pili (Fig. 4C). To determine if the Csu pili were involved, their CsuA/B and CsuE subunits were monitored using immunofluorescence staining. The confocal laser microscopy analyses clearly demonstrated that the bacteria adhering to the epithelial cells expressed the CsuA/B subunit (Fig. 4D) and the tip protein CsuE (Fig. 4E). Taken together, these findings suggest that Csu pili mediate the adherence of *A. baumannii* to A549 epithelial cells. To further assess the impact of the Csu pili on the invasion of epithelial cells, we performed a gentamicin protection assay to quantify intracellular bacteria. The invasion assay revealed that 72.2 ± 12.3% of cell-associated bacteria adhered to the surface of the cell lining, while the rest resided intracellularly. Interestingly, intracellular bacterial count was slightly increased in the *csuA* mutant as compared to the wild type (Fig. 4F).

Considering the observation that the expression of Csu pili is tightly regulated by growth conditions (Fig. 2D, Supplementary Fig. 1B), we further examined the adherence and invasion phenotypes of *A. baumannii* AB17978 and the *csuA* mutant cultured in LB agar plates, a condition in which CsuA/B expression was not detected (Fig. 2D). The adherence capability of the *csuA* mutant was not significantly altered as compared to wild type (Fig. 4G) as reported previously for *A. baumannii* 19606^18^ and its *csuE* mutant derivative. However, the invasion of epithelial cells by the *csuA* mutant was significantly increased as compared to the wild type (Fig. 4H). Taken together, these results suggest that the involvement of the Csu pili is confined to colonization on the surface of the epithelial lining, while they have a negative impact on the bacterial ability of invasion.

Since *A. baumannii* causes a wide variety of infections and also resides as an aseptic carrier in the gastrointestinal tract^40,41^, we further tested the interactions between SW480 epithelial cells derived from human rectal carcinoma and *A. baumannii* AB5075 as well as other clinical isolates. While all tested *A. bumannii* clinical isolates adhered to SW480 cells, their ability to adhere varied among the different isolates. Noteworthy, we did not detect invasion of SW480 cells with *A. baumannii*. The adherence capability of the *A. baumannii* strain Ab-Pak-Lah-14 was almost twice as high as that of the AB5075 strain. Consistent with this adherence potential, the expression level of the Csu pili was found to be higher in the case of the Ab-Pak-Lah-14 strain than in strain AB5075 (Fig. 3B). We also measured to what extent the *A. baumannii* clinical isolates caused induction of the cytokine IL-8 from SW480 epithelial cells (Fig. 4J). Strain Ab-Pak-Lah-14 induced a considerably higher IL-8 level than strain AB5075, and evidently, the pattern of IL-8 induction correlated with the adherence potential of the clinical isolates.

### The interplay of biofilm formation and virulence mediated by Csu pili is regulated by the GGDEF-EAL protein PdeB

**Fig. 5:**
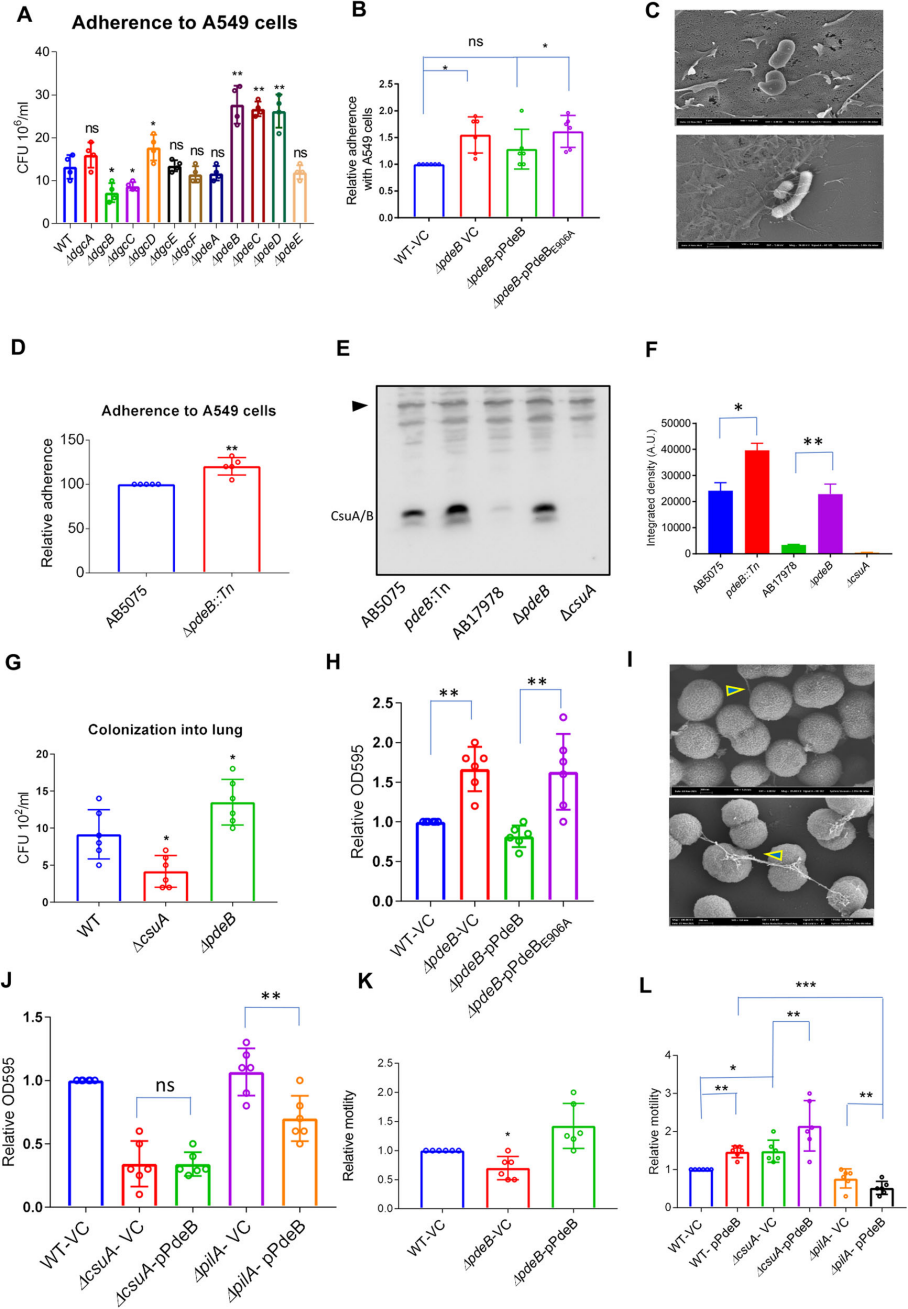
GGDEF/EAL protein AIS_2337 is a modulator of Csu-dependent adherence and biofilm formation and affects PilA-dependent motility of *A. baumannii* 17978. **A** Bar chart diagrams illustrating the screening of the individual mutants of 11 GGDEF/EAL proteins of *A. baumannii* 17978 for adherence to epithelial cells A549. **B** Adherence of *pdeB* mutant and trans-complemented strains with epithelial cells A549 in comparison with wild-type 17978 vector control (WT-VC). The statistical significance of the difference between vector control and trans-complemented strains was measured by a non-parametric *t* test using Graph Pad Prism. **C** Scanning electron microscopic images of *A. baumannii* 17978 wild-type (Upper panel) and *ΔpdeB* mutant (Lower panel) bacteria attached to A549 epithelial cells, highlighting the expression of pili appendages on the *ΔpdeB* mutant strain. **D** Bar chart diagram to demonstrate the comparative analysis of the adherence capability of *A. baumannii* AB5075 and *pdeB::Tn* mutant strains grown in LB broth over night at 30 °C to the epithelial cell line A549. **E** Western blot analysis of *pdeB* mutants in *A. baumannii* 17978 and AB5075 backgrounds for the expression of CsuA/B. The samples were collected from static cultures grown for 72 h in LB without salt at 30 °C. **F** Bar chart diagram showing the average integrated density of CsuA/B bands from three blots. **G** Bar chart diagrams showing relative colonization of bacteria into the lungs of BALBc mice sacrificed after 24 h of internasal infection and measured upon CFU counting of bacterial colonies on LB agar plates. CFU counts shown here represent CFU per millilitre of homogenized tissue. **H** Bar chart diagram showing relative levels of biofilm formation by the following *A. baumannii* 17978 strains: the wild-type, the *ΔpdeB* mutant and the trans-complemented mutant with wild type and catalytic mutant proteins at 30 °C after 72 h of incubation in microtiter plates and measured as the optical density of biofilm matrix stained with crystal violet. **I** Scanning electron microscopic images of *A. baumannii* 17978 wild-type (upper panel) and *ΔpdeB* mutant (lower panel) bacteria from cultures in LB at 30 °C after 72 h of incubation in glass tubes. **J** Bar chart diagram showing relative levels of biofilm formation by *ΔcsuA* and *ΔpilA* mutant strains and effect of the overproduction of PdeB from the plasmid pMBB67 (pPdeB). **K** Bar chart diagram of surface-associated motility assessed by measurements of the increased zone diameter *of A. baumannii* 17978 wild type (WT) and *ΔpdeB* mutant harbouring pMBB67EH vector control after 6 h at 37 °C. **L** Bar chart diagram of surface motility assessed by measurements of the increase in the zone diameter of *ΔcsuA* and *ΔpilA* mutant strains and effects of the overproduction of PdeB. Statistical significance of the difference shown as *p* value that was measured by non-parametric two tailed *t* test. A single asterisk * indicates *p* ≤ 0.05, two-asterisks ** indicate *p* ≤ 0.01, three-asterisks *** indicates *p* ≤ 0.001 and “ns” indicates non-significant difference.

The major involvement of the Csu pili in mature biofilm development and virulence prompted us to study the upstream signalling pathway involved in Csu-mediated virulence. We have recently characterized a network comprising eleven proteins harbouring GGDEF/EAL domains involved in the c-di-GMP homeostasis of *A. baumannii* 17978^42^. The six GGDEF proteins encoded by loci *AIS_0751, AIS_1067, AIS_1695, AIS_3296, AIS_2986* and *AIS_2506*, here annotated as DgcA, DgcB, DgcC, DgcD, DgcE and DgcF, respectively, were potential diguanylate cyclases^42^. The confirmation of diguanylate cyclase activity of these six proteins, along with an additional GGDEF protein AIS_3345, has been recently demonstrated biochemically^43^. The three GGDEF/EAL proteins encoded by loci *AIS_2337, AIS_0546* and *AIS_1949*, here referred to as PdeB, PdeD and PdeA, respectively, were described as potential c-di-GMP phosphodiesterases based on bioinformatic and phenotypic analysis of these proteins. The EAL protein encoded by locus *AIS_1254*, here annotated as PdeC, was a potential phosphodiesterase and the EAL protein encoded by locus *AIS_2422*, here annotated as PdeE, was considered enzymatically inactive^42^ (Table 1). Consistently, the phosphodiesterase activity of these four proteins has been proven biochemically^43^. To test for a potential involvement of c-di-GMP signalling in the Csu-mediated virulence, we utilized a set of single deletion mutants of all the GGDEF/EAL loci in *A. baumannii* 17978 and we used cultured A549 cells to evaluate the mutants for adhesion. The mutants defective in the GGDEF/EAL proteins DgcB and DgcC showed significantly decreased adhesiveness, suggesting that those proteins may act as enhancers of the adherence phenotype. The mutants deficient in the PdeB, PdeC and PdeD proteins showed enhanced adhesiveness, indicating that they as well may act as adherence phenotype modulators (Fig. 5A). A deletion in the gene coding for the GGDEF/EAL protein PdeB was found to have the greatest impact on the adherence phenotype. Furthermore, the effect of the *ΔpdeB* mutation was similar when the adherence ability of *A. baumannii* was tested with SW480 epithelial cells, although a different panel of GGDEF/EAL proteins appear to influense adherence to SW480 cells (Supplementary Fig. 4A, B). PdeB alias AIS_2337 is a catalytically active phosphodiesterase harbouring a catalytically inactive GGEDEF domain^43^ with additional PAC and PAS domains (Supplementary Fig. 4C). The phosphodiesterase activity of PdeB is abolished upon replacing glutamate 906 with alanine from EGVE motif of the protein as shown in rdar (red dry and rough) morphotype model of *S. typhimurium* SR-11 (Supplementary Fig. 4C). The suppression of rdar morphotype is linked to phosphodiesterase activity of EAL proteins.

**Table 1:** GGDEF/AL domain proteins in *A. baumannii* 17978.

*Annotation of GGDEF/EAL domains in A. baumannii*	*Gene loci*	*GGDEF/EAL domain/motif*
DgcA	*AIS_0751*	GGEEF
DgcB	*AIS_1067*	GGEEF
DgcC	*AIS_1695*	GGEEF
DgcD	*AIS_2506*	GGDEF
DgcE	*AIS_2986*	GGDEF
DgcF	*AIS3296*	GGDEF
PdeA	*AIS_1949*	GGDEF-EAL
PdeB	*AIS_2337*	GGDEF-EAL
PdeC	*AIS_1254*	EAL
PdeD	*AIS_0546*	GGDEF-EAL
PdeE	*AIS_2422*	QIV

The *trans*-complemented expression of the PdeB protein restored the wild-type adherence phenotype. However, the catalytic mutant of PdeB annotated as PdeB_E906A_ could not restore a wild type level adherence of the *pdeB* mutant (Fig. 5B) suggesting that the phosphodiesterase activity per se of PdeB is required for its effect on adherence of *A. baumannii* to A549 epithelial cells. SEM analyses of the *ΔpdeB* mutant *A. baumannii* 17978 attached to A549 epithelial cells suggested that it might occur via the expression of long pili, i.e. the Csu pili (Fig. 5C). The adherence of the *A. baumannii* AB5075 *pdeB* transposon mutant to A549 cells was also significantly increased as compared to the wild type strain (Fig. 5D). Western blot analyses of *pdeB* mutant derivatives of both (AB5075 and AB17978) tested strains revealed enhanced expression of CsuA/B in the *pdeB* mutant strains (Fig. 5E, F). Next, we tested the virulence of *pdeB* and *csuA* mutants upon intranasal infection of BALB/c mice. Mice were infected intranasally with bacteria and sacrificed after 24 h to estimate bacterial colonization in the lung tissue. Although mice did not show apparent physical signs and symptoms of infection, viable bacteria were detected in the lungs of sacrificed mice (Fig. 5G). The number of CFU/ml was significantly decreased in mice infected with the *csuA* mutant compared to mice infected with the wild type, whereas the bacterial number in mice infected with the *pdeB* mutant was significantly increased (Fig. 5G). These findings suggest that colonization of the lung tissue by *A. baumannii* is inversely influenced by PdeB and Csu pili.

Next, we investigated the effect of the *ΔpdeB* deletion mutant of AB17978 on biofilm formation and on bacterial motility as monitored by migration zone diameter. The *ΔpdeB* mutant showed increased biofilm formation in glass tubes at 30 °C (Fig. 5H). The expression of PdeB from plasmid suppressed the biofilm formation in the *pdeB* mutant whereas PdeB_906_ variant did not alter biofilm formation. This finding suggests that PdeB suppresses biofilm formation through phosphodiesterase activity of its EAL domain. Furthermore, SEM analysis of the *ΔpdeB* mutant strain verified the expression of long pili in this mutant (Fig. 5I). The overexpression of PdeB from a plasmid cloned in the *csuA* mutant did not alter the mutant´s defect in biofilm production, but it suppressed the biofilm formation of the *pilA* mutant strain (Fig. 5J). These findings suggest that PdeB inhibits biofilm formation through a Csu-dependent pathway.

*A. baumannii* can move on the surface of a semisolid medium^44^. A stereomicroscopic image of the motility zone illustrates that *A. baumannii* moves across soft agar in uniformly distributed leaf-like patterns (Supplementary Fig. 6). The mutant lacking PdeB exhibited reduced motility with non-uniformed patterns as compared to the wild type (Fig. 5K and Supplementary Fig. 6). The expression of PdeB from the plasmid restored the motility defect of the *ΔpdeB* mutant strain (Fig. 5K). In contrast, PdeB expression from the plasmid did not cause increased motility of the *ΔpilA* mutant, instead the motility was reduced (Fig. 5L). Although the *ΔpilA* mutant did not exhibit alterations in surface-associated motility, the in-trans overexpression of PilA from a plasmid resulted in the development of uneven margins in the motility pattern, similar to that of the Δ*pdeB* mutant (Supplementary Fig. 6). The surface motility of the *ΔcsuA* mutant was found to be significantly increased as compared to the wild type (Fig. 5L). These findings suggest that the Csu pili suppress the motility of *A. baumannii* whereas the impact of PdeB on motility is independent of Csu pili. Altogether, the data suggest that PdeB regulates motility and biofilm formation of *A. baumannii* by distinct pathways.

### Both PdeB and Csu pili influence virulence in a mouse model of intraperitoneal infection

**Fig. 6:**
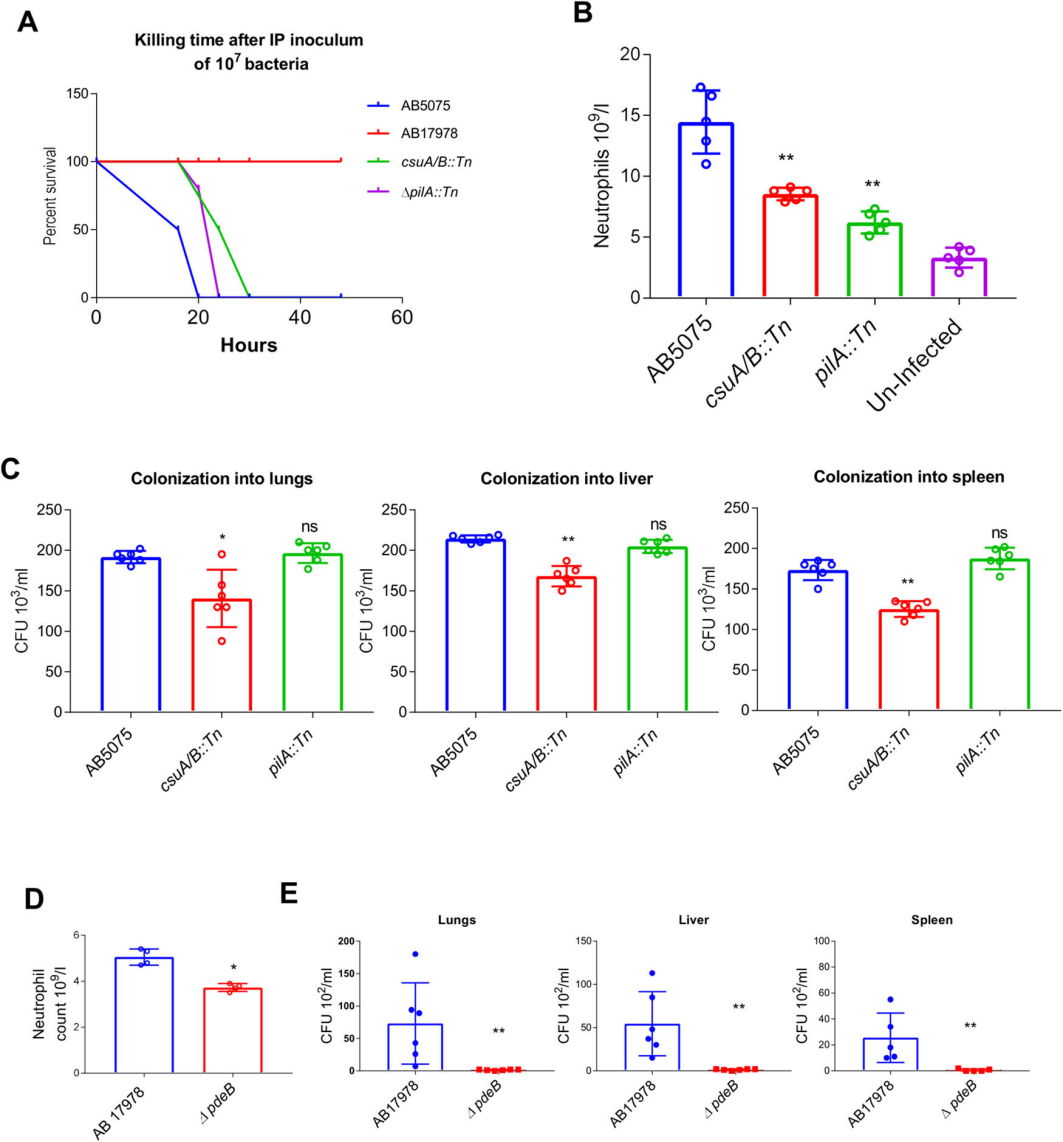
Influence of Csu pili, type IV pili, and AIS_2337 on systemic spread of intraperitoneal infection in an experimental mouse model. **A** The plotted data show the percentage of mice that survived at different time points after intraperitoneal infection (IP) using the inoculum dose of 10^7^ bacteria per mouse. In total, 6 mice were infected with each isolate. **B** Bar chart diagram showing total neutrophil count in the blood withdrawn through heart puncturing from mice after 18 h of infection with wild type cells and transposon insertion mutants of the hypervirulent strain AB5075. **C** Bar chart diagrams showing relative colonization of bacteria into lungs, liver and spleen of mice, sacrificed after 18 h of intraperitoneal infection and measured upon CFU counting of bacterial colonies on LB agar plates. CFU counts shown here represent CFU per millilitre of homogenized tissue. Each experiment was performed with 6 mice. **D**, **E** Effect of the *ΔpdeB* mutation on systemic infection by the low virulent *A. baumannii* strain 17978 in the BALBc mouse model using an inoculum dose of 10^7^ bacteria per mouse for intraperitoneal infection. **D** Bar chart diagram showing total neutrophil count in blood of mice infected with *A. baumannii* 17978 wild type and *ΔpdeB* mutant, using samples withdrawn after 18 h. **E** Bar chart diagram showing the relative colonization of bacteria in the lungs, liver, and spleen of mice after 18 h of intraperitoneal infection and measured upon CFU counting of bacterial colonies on LB agar plates. Each experiment was performed with 6 mice. Error bars show mean ± SD. Statistical significance is indicated by *p* value that was measured by non-parametric two tailed *t* test. A single asterisk * indicates *p* ≤ 0.05, two asterisks ** indicate *p* ≤ 0.01 and “ns” indicates non-significant difference.

AB5075 and AB17978 strains were further tested for their virulence potential in a murine model of acute intraperitoneal infection. An inoculum of 10^7^ cells of the AB5075 wild-type strain was lethal to mice within 19 (+/–2, 3) hours after intraperitoneal infection (Fig. 6A). In contrast, all the mice infected with a similar dose of the less virulent AB17978 strain survived the infection. This finding is consistent with the established fact that AB5075 is a hyper-virulent strain of *A. baumannii*^45^. However, *pilA* or *csuA/B* transposon insertion mutants required longer killing times compared to the wild-type AB5075 strain. The time required by the *csuA/B* mutant or *pilA* mutant strains to kill infected mice was 30 (+/–2) and 27 (+/–2) hours, respectively. After 18 h of infection, the total neutrophil count in the blood of mice infected with the *csuA/B* or *pilA* mutants was significantly lower than that of mice infected with the wild-type strain (Fig. 6B). Moreover, the bacterial colonization in the lungs, liver, and spleen was decreased in mice infected with the *csuA/B* mutant but not in mice infected with the *pilA* mutant (Fig. 6C). These findings suggest that Csu pili contribute to the virulence and spread of the infection in this mouse model.

We used the less virulent *A. baumannii* 17978 strain to assess the effects of the *ΔpdeB* mutation on virulence in the same mouse model. After 18 h of infection, the neutrophil count in the blood of mice infected with the *ΔpdeB* mutant was significantly lower than that of the animals infected with the wild-type *A. baumannii* 17978 (Fig. 6D). In addition, there was little to no colonization of *ΔpdeB* mutant bacteria in the lungs, spleen and liver, while the *A. baumannii* 17978 wild-type bacteria were found at relatively high numbers in these organs (Fig. 6E). These findings suggest that the EAL protein PdeB plays a pivotal role in the virulence of *A. baumannii*.

## Disucussion

The elucidation of the role of Csu pili in adherence to epithelial cells is a key finding that enhances our understanding of the initial event of host pathogen interaction during human infection with *A. baumannii*. Here, aided by genetic analysis and several microscopic techniques, we presented the evidence for the involvement of the Csu pili in the attachment to epithelial cells (Fig. 3). Pathogenic bacteria are equipped with multiple types of pili that can be used to interact with their host^36–39^. Although Csu pili induce a specific protective immune response against *A. baumannii* in a mouse model, their functional roles in the virulence phenotypes of *A. baumannii* have been less clear^46^. The adherence of *A. baumannii* to epithelial cells is a strain-specific trait that varies significantly from one strain to another^17^. The growth conditions and the type of epithelial cells account for an additional level of variability. Here, we demonstrate that the expression of Csu pili is one of the determinants of this variability during epithelial cell adhesion. Our findings further clarify that Csu pili are primarily responsible for surface colonization rather than invasion of epithelial cells.

Regarding the role in virulence of Csu pili, we have examined the consequences of altered Csu pili expression for *A. baumannii* intraperitoneal infection and intranasal infection using two different mouse models (Fig. 5G and Fig. 6A-G). Upon infection with *csuA* and *pdeB* mutant strains, the results from both models appear to be notably different. *A. baumannii* lacking Csu pili appear to lose the ability to colonize body organs, as observed in both models. In contrast, the AB17978- *pdeB* mutant with increased capability of CsuA/B expression colonised lung tissue more efficiently when administered via the intranasal route, despite being attenuated when administered intraperitoneally. These findings suggest that CsuA/B pili are not required to breach the epithelial lining during systemic spread from intraperitoneal infection, but they contribute to virulence by facilitating surface colonization. In accordance with this finding, the biofilm formation capability was intrinsically higher in case of *A. baumannii* isolates that caused lung infection compared to isolates responsible for bacteraemia^47^. Even the expression of Csu pili by genetically engineered *E. coli* enhanced the bacterial ability to adhere to lung epithelial cells^32^. Csu pili are equally important to build interactions not only with host cells but also among bacterial cells to form mature biofilms. Csu assembly-deficient mutants of both AB17978 and AB5075 were incapable of mature biofilm formation in glass tubes, on plastic surfaces and in plastic growth chambers and of pellicle formation (Figs. 1 and 2). It has been shown recently that the cell density dependent formation of microscopically visible mushroom-shaped biofilm structures within rolling biofilm bioreactors also require the Csu pili^29^. The mushroom-shaped dense biofilm structures formed in rolling biofilm reactors represent biofilm formation at an air-liquid interphase. However, in the current experimental settings of 18-well chamber glass slides, *A. baumannii* 17978 did not form such mushroom-shaped structures. Instead, the bacteria formed microscopically visible structures, here denoted as “mountain-like” biofilm patches. Presumably, the formation of such different structures would be due to the absence of an air liquid interphase on the bottom of the glass well.

By employing multiple experimental settings, we defined the role of Csu pili in biofilms and visualized their precise location in mature biofilm patches. The temporal and spatial arrangement of cells equipped with Csu pili on the top of mountain-like patches evidently provided protection against the bactericidal effects of antimicrobial agents as demonstrated with the last line antibiotic colistin (Fig. 3D). Hence, the protective lining of Csu pili-enriched bacterial cells on the surface of mature biofilms would play an important role in adaptive drug resistance. *A. baumannii* grown within biofilms can be tolerant also to other antibiotics as shown with kanamycin^29^. Altogether, the present findings together with recently published additional evidence^29,48^ suggest that the expression of Csu pili would be suitable potential target in efforts to prevent and manage the adaptive drug resistance in *A. baumannii*. In this regard, subinhibitory concentrations of trimethoprim and sulfamethoxazole were shown to suppress Csu pili expression and can be considered candidates as therapeutic agents to manage adaptive drug resistance in *A. baumannii*^48^.

Notably, the expression of Csu pili and their contribution to biofilm formation is detected in later stages of bacterial growth (Fig. 1A) whereas the expression of type IV pili was restricted to the early stages of bacterial growth^49^. Although the focus of this study mainly remains to investigate expressional and functional features of Csu pili and the expression of type IV pili remains undetermined here, our data together with the findings presented previously^49^ suggest that growth conditions favour the expression of one type of pili over the other. In contrast to type IV pili, the expression of Csu pili appears cells density-dependent as shown with its increased expression over an increase in the density of cultures (Fig. 1A). The formation of mushroom-shaped biofilm structures is linked to the AHL synthase AbaI in *A. baumannii* 17978^29^. Hence, we suggest that the formation of mountain-like biofilm patches shown here is also regulated by quorum sensing as individual bacteria hardly expressed any Csu pili outside the biofilm patches (Supplementary Fig. 2A). Csu pili were not required to initiate the adherence to the abiotic glass surface but contributed to the maturation of biofilms. A similar distribution of task in biofilm formed by two types of pili is shown in the case of *Salmonella typhimurium*. The initial surface adherence of bacteria was achieved through type 1 pili, whereas curli fimbriae contributed to the maturation of biofilm development after 48 h^50^. The Cup pili of *Pseudomonas aeruginosa*, encoded by genes homologous to the *csu* operon of *A. baumannii*, were shown to be responsible not only for the adherence to abiotic substrates but also for cell clustering and the formation of mushroom-shaped structures during biofilm growth^51^. Similarly, additional chaperone/usher paralogues in *P. aeruginosa*, such as the pilus assembly proteins CupB and C, facilitate cell-to-cell interaction and contribute to the biofilm maturation process^52^.

The promoter region of the *A. baumannii csu* operon appears to be a hub of several kinds of transcriptional regulators as judged by our inspection of the DNA sequence (Supplementary Fig. 6). Potential binding sites for response regulator PhoP, oxygen response transcriptional regulators Fnr and DcuR, amino acid biosynthesis regulators LeuO and ArgR, replication initiator protein DnaA, and HTH-AraC-like transcriptional regulator are found in a 583-bp promoter region. Most interestingly, there are binding sites for an HTH-CRP-like transcriptional regulator on both trans and cis DNA strands within the promoter region. A divergently transcribed HTH-type transcriptional regulator with locus ID AIS_3796 co-exists in connection with this Csu promoter region. Based on this orientation of AIS_3796, and the presence of potential binding sites in both directions in the promoter region, we speculate that AIS_3796 may regulate expression of the *csu* operon through self-limited feedback inhibition. The occurrence of apparent binding sites for multiple transcriptional regulators at the *csu* promoter region enable us to consider that Csu pili can serve multiple roles for *A. baumannii*. This promoter can be seen as a check point for differential expression of Csu pili and optimal regulation of biofilm formation and virulence phenotypes. However, further insights into regulatory and signalling pathways are required in order to better understand the mechanisms behind such adaptive features.

The GGDEF-EAL protein PdeB appears to play a central role in the regulation of biofilm formation, motility and adherence to epithelial cells (Fig. 5). PdeB is a GGDEF/EAL protein with an N-terminal transmembrane domain and two PAC domains spanning a PAS domain^42^. Protein blast searches identified that PdeB is a GGDEF/EAL protein unique to *A. baumannii* with no apparent homologs currently described. Although the AIS_2337 mutant of *A. baumannii* 17978 could adhere to epithelial cells, it appeared to be less virulent in terms of causing systemic infection (Fig. 6D, E). The increased expression of Csu pili in this mutant enhanced the colonisation of bacteria on the epithelial lining. Perhaps these long pili connect several bacterial cells, and if such bacterial aggregations can successfully adhere to the epithelial cell lining, then it might cause trapping of bacterial cells while preventing bacterial spreading into systemic infection. However, a more comprehensive investigation is necessary to experimentally test this hypothesis. Also, in *P. aeruginosa*, the EAL- domain-containing PvrR supresses the formation of Cup pili assembly by inhibiting the expression of *cupD* indirectly through the response regulator RcsB. This function of PvrR is suggested to require c-di-GMP degradation through phosphodiesterase activity, supporting the role of c-di-GMP in the regulation of Cup pili-mediated biofilm formation^53^. The homologue of RcsB exists in *A. baumannii* at the locus SCY56367.1. Therefore, the involvement of EAL protein PdeB, alias AIS_2337, in preventing Csu-mediated biofilm formation indicated that a similar c-di-GMP regulatory mechanism can exist in *A. baumannii*.

The motility of *A. baumannii* has appeared somehow enigmatic since this organism lacks a flagellum and is immotile in terms of typical swimming or swarming. On the other hand, this bacterium has been shown to exhibit surface-associated motility as well as twitching motility. The mechanisms behind surface mobility are very poorly understood^20^. Multiple regulators, such as purine and pyrimidine biosynthetic machineries, quorum sensing, natural competence, and c-di-GMP signalling, are known to be involved in surface-associated motility^54^. This observation on a cross regulatory regulation of different kinds of pili in *A. baumannii* opened an interesting line of research for follow up investigations (Supplementary Fig. 2). Based on findings in *P. aeruginosa*^55–57^, we can speculate that the loss of PilA makes the bacteria more mechanosensitive, since the distribution of the mechano sensor PilY1 (which exists as a homologue in *A. baumannii* with locus tag SSV46524) is cytoplasmic, periplasmic and on the outside leaflet of the outer membrane of the PilA-deficient strain. Such increased mechano sensitivity can trigger the synthesis of c-di-GMP via SadC-like cyclase, which interacts with the T4P alignment complex. Consequently, as we show here, high levels of c-di-GMP promote the expression of the Csu fimbriae.

**Fig. 7:**
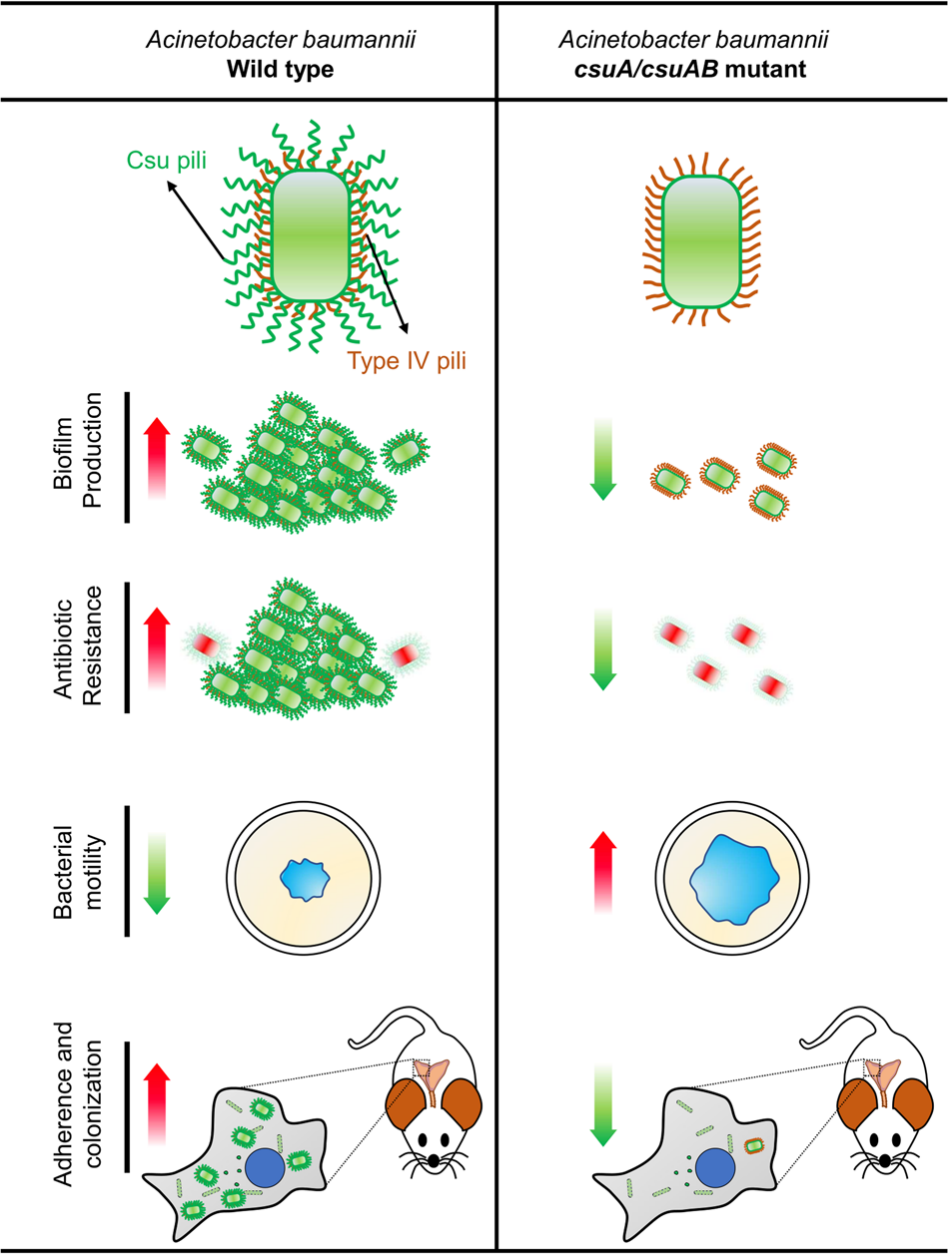
Schematic diagram to compare *A. baumannii* wild type with its Csu pili deficient variant (*csuA* and *csuA/B* mutants) for different phenotypes. Csu pili promote biofilm formation, colistin tolerance, adherence to epithelial cells and colonization into the lung, liver, and spleen in a mouse model, whereas it inhibits bacterial motility. Red arrow represents increased activity, whereas green arrow represents reduced activity.

Taken together and summarized in Fig. 7, our data suggest that there is an interplay between the Csu pili and type IV pili depending on the environmental conditions and c-di-GMP-metabolizing proteins, which modulate motility-biofilm behaviour/transitions as well as host surface colonization, influencing the systemic infection properties of *A. baumannii*. Being a central player in regulation of biofilm formation, adaptive drug resistance, cytokine induction and virulence phenotypes, Csu pilus assembly and function are proposed to be suitable targets for the development of new therapeutic strategies to treat and prevent infections caused by multi-drug resistant *A. baumannii*.

## Methods

### Bacterial strains and growth conditions

All the bacterial strains used in this study are listed in Supplementary Table 2. Strains were stored in a 15% glycerol solution in LB broth at −80 °C. The growth of *A. baumannii* strains was maintained in LB or on LA plates. YT (Tryptone Yeast Extract) broth was used for *Escherichia coli* in the procedure for plasmid construction and for the generation of *A. baumannii* deletion mutants, as described below. In order to maintain plasmid constructs of the vector pMMB67EH, 100 µg/ml carbenicillin was supplemented with the media. 1 mM Isopropyl β-D-1-thiogalactopyranoside (IPTG) was used to induce expression of the cloned genes in pMMB67EH.

### Plasmid constructions

The plasmids used in this study are listed in Supplementary Table 3. Oligonucleotide primers used are described in Supplementary Table 4. The genes encoding PilA and CsuA proteins of *A. baumannii* 17978 were cloned into the broad host vector pMMB67EH^58^ as C-terminal 6xHis fusion constructs. DNA sequences, including open reading frames along with the ribosomal binding region of individual genes, were amplified using template DNA isolated from *A. baumannii* 17978 and Phusion Polymerase (Thermo Scientific). The oligonucleotide primers contained sequences for suitable restriction endonuclease cleavage sites and a combination of *Sac*1/*Bam*H1 endonucleases was used to digest the PCR product. Digestion products were purified with the Gene Clean kit (Thermo Scientific) and ligated with DNA of the pMMB67EH plasmid vector digested with the same combination of *Sac*1/*Bam*H1.

For each construct, the ligation mixture was introduced into chemo-competent *E. coli* DH5α by heat shock treatment at 42 °C for 1 min, with subsequent growth in 2x YT broth at 37 °C for 1 h. The transformants of *E. coli* were selected on agar plates containing carbenicillin (100 μg/ml) and confirmed by PCR using a forward primer upstream and a reverse primer downstream of multiple cloning sites. The integrity of the constructs was confirmed by DNA sequencing of the inserted genes, performed by Eurofins GATC Biotech, Germany.

### Construction of PdeB_E906A_

To generate E906A mutation in the EAL domain of PdeB, Quick Change mutagenesis kit (Agilent Technology) was used. pMBB67EH harbouring PdeB was used as a template. Mutagenic oligonucleotides used in this experiment are listed in table S3. The resulting mutation was confirmed by DNA sequencing.

### Construction of mutant strains of *A. baumannii* 17978

Chromosomal deletion mutants of the *pilA, csuA, csuC* genes and the genetic determinants of the eleven different genes for GGDEF/EAL proteins in *A. baumannii* 17978 were generated in a one-step gene replacement by homologous recombination^59^. The entire open reading frames, except 100 nucleotides at the beginning and at the end of the gene, were replaced by a kanamycin resistance marker allele /gene. The kanamycin gene, along with sequences corresponding to the target gene such that there were homologous overhangs of 100 bps, was PCR‐amplified from plasmid pKD4 and introduced by electroporation into *A. baumannii* carrying the plasmid pAT02, a derivative of pMMB67EH where a gene for the recombinase RecAB is inserted. Prior to electroporation, *A. baumannii* carrying pAT02 was grown to log phase in LB containing carbenicillin (100 μg/ml) and IPTG (2 mM). After 3 washes with ice-cold 10% glycerol and subsequent 1000-fold concentration of the bacterial sample, 100 µl of bacterial suspension (~10^10^ bacteria) were mixed with 5 µg of recombinant PCR product and electroporated in a 2-mm cuvette at 1.8 kV, Ohm. After recovery in 4 ml of YT medium containing 2 mM IPTG, the bacteria were pelleted, plated on LB-agar with 7.5 µg/ml kanamycin, and incubated overnight at 37 °C. All constructed mutants were verified by PCR with control primers matching sequences in the genes flanking the deleted open reading frame.

### Scanning electron microscopy

For scanning electron microscopy (SEM), five microliters of bacterial suspension in phosphate buffered saline (PBS) at a cell density of OD_600_ = 1 from an overnight plate culture were spotted onto LB agar plates. The plates were incubated at 37 °C for 24 h. Pieces of agar containing bacterial colonies were collected for imaging. In the case of air-liquid interphase, bacterial pellicles were collected with a plastic loop. For SEM of *A. baumannii* infected epithelial cells, A549 lung carcinoma cells were grown on glass cover slips (60,000 cells per well) in a 24-well plate and infected with bacteria at a multiplicity of infection (MOI) 1:100 during four hours. All the samples were fixed overnight at 4 °C with 2.5% glutaraldehyde in 0.1 M phosphate buffer, and subsequently dehydrated in a graded series of ethanol, critical point dried, and coated with 5 nm palladium. The bacterial cell morphology was analyzed by imaging with a field-emission scanning electron microscope (Carl Zeiss Merlin FESEM) using secondary electron detectors at an accelerating voltage of 4 kV, and a probe current of 50–120 *p*A.

### Biofilm formation assay in glass tubes

Biofilm formation assays were performed in sterilized glass tubes. Bacterial cells grown overnight on LA plates were suspended in PBS at an OD_600_ = 1. From this suspension, 200 µl were added to each tube containing 3 ml of LB without salt supplemented with 100 μg/ml carbenicillin with or without 1 mM IPTG. Tubes were incubated statically at 30 °C or 37 °C for 72 h. Subsequently, the liquid contents were discarded, and the tubes were washed gently with water. Bacterial cells attached to walls, either at the air-liquid interphase or at the base in the form of biofilm, were stained with 1% crystal violet for 30 min. Excess crystal violet was removed by washing three times with water. Cells stained with crystal violet were dissolved in 1 ml of 5% acetic acid solution. The intensity of the crystal violet colour represents the abundance of biofilm, and it was measured as optical density with a 595 nm filter. Data were subjected to statistical analysis using Graph Pad Prism software.

### Analyses of biofilm patches formed on a glass surface

For analyses of biofilm morphology and selected biofilm components, the assays were performed on sterilized 18-well chamber glass slides (Ibidi GmbH). Bacterial cells grown overnight on LB agar plates were suspended in PBS to an OD_600_ = 1. From this suspension, 20 µl of bacterial suspension were added to each well, containing 180 μl of LB without salt broth supplemented with 100 μg/ml carbenicillin with or without 1 mM IPTG. Chamber slides were incubated in a moist chamber at 30 °C for 72 h. Subsequently, the liquid contents were removed, and the plates were washed gently with PBS. Bacterial cells attached to the glass surface in the form of biofilm were stained with Hoechst 33342 (2 μM). Components of the biofilm were investigated by staining bacterial biofilm with Alexa 647-labelled Wheat Germ Agglutinin (Alexa647WGA, 20 μg/ml). For detection of Csu pili in the biofilm, bacterial biofilm was fixed with 4% paraformaldehyde (PFA), permeabilized with triton X-100 (0.25 %), followed by incubation with CsuA/B anti-serum (1:100 dilution in 5% FCS/PBS) at 4 °C overnight. After washing with PBS, CsuA/B was detected with goat anti-rabbit secondary antibodies conjugated to Alexa488 or Alexa555 (1:200 dilution, 1 h at room temperature (RT). Biofilms were quantified using the COMSTAT^60^ plugin in ImageJ^61^. For quantification of biofilm using COMSTAT, images were processed using an automatic thresholding method build in the graphical user interface (GUI) of COMSTAT 2.1, followed by the calculation of total biomass and average biofilm thickness.

### Assay for surface-associated motility

Surface-associated motility assays were performed in petri plates containing soft agarose medium, as described previously^20^. Soft agarose media consists of tryptone (5 g/l), agarose (5 g/l) and sodium chloride (2, 5 g/l). For the motility assay, five microliters of bacterial suspension in PBS (OD_600_ = 1) from an overnight plate culture were spotted onto soft agarose plates supplemented with 100 µg/ml carbenicillin with or without 1 mM IPTG. The plates were incubated at 37 °C for 7 h. The diameter of motility zones was recorded and analyzed using Graph Pad prism software.

### Rdar morphotype assay

Five microliters of a bacterial suspension in PBS (OD_600_ of 5) from an overnight culture in LB broth were spotted onto LB without salt agar plates supplemented with Congo red (40 μg ml^−1^), Coomassie brilliant blue (20 μg ml^−1^), carbencillin (100 μg ml^−1^) and IPTG (1 μM). Plates were incubated at 30 °C for 24 h. Development of the colony morphology and dye binding were analysed after 24 h.

### Western immunoblotting analysis

Western immunoblotting analysis for CsuA/B in biofilm samples from pellicles was performed essentially as described previously^23^ with slight modifications. Briefly, overnight bacterial cultures were grown in different conditions as described in legend of respective blot. Bacteria in the air-liquid interphase formed a pellicle. Cells recovered from the pellicle were mixed with Laemmli buffer and boiled for ten minutes. The proteins were separated by electrophoresis in 15% SDS polyacrylamide gels and transferred for immunoblotting onto a polyvinylidene difluoride membrane (Bio-Rad Laboratories) in Bio-Rad A-buffer (25 mM Tris, pH 8.3, 192 mM glycine, 20% methanol, and 0.1% SDS) at 100 V for 1 h. Membranes were blocked with 5% skimmed milk in PBS/Tween, incubated with a primary anti-CsuA/B rabbit polyclonal antibody (Innovagen AB)^62^, followed by incubation with a secondary HRP-conjugated anti-rabbit goat antibody (AgriSera AB, Sweden Cat# AS09602) with 1:5000 dilution in % skimmed milk in PBS/Tween. Chemiluminescence (Lumi-Light WB substrate; Roche) was visualized using the LAS-1000 system (FUJIFILM). The full unedited images of immuno blots generated by LAS-1000 system are provided in the Supplementary Fig. 7.

### Atomic force microscopy

*A. baumannii* cells were imaged by Atomic Force Microscopy (AFM) as described previously^63^. Briefly, *A. baumannii* recovered from plate grown colonies or from pellicles were washed in PBS and placed onto freshly cleaved mica (Goodfellow Cambridge Ltd., Cambridge, United Kingdom). The samples were blot-dried and desiccated prior to imaging. Imaging was done on a Multimode 8 Nanoscope AFM equipment (Digital Instruments, Santa Barbara) using Tapping Mode TM. A silicon probe oscillated at its resonant frequency of approximately 300 kHz, selected by the Nanoscope software. Images were collected in the air at a scan rate of 0.8–1.5 Hz, depending on the size of the scan and the number of samples (256 or 512 samples/image). The final images were plane-fitted in both axes and presented in a surface plot of the height mode.

### Confocal microscopy

Lung carcinoma cells, A549 were seeded in 8-well chamber slides (3 × 10^4^), in RPMI-1649 complete media, overnight at 37 °C and 5% CO_2_. The following day, cells were infected with *A. baumannii* (MOI 1:100) for 4 h. The non-adhered bacteria from infected cells were washed with PBS (three times). *A. baumannii* adhered to A549 cells were fixed with 4% PFA, permeabilized with triton X-100 (0.25 %), followed by incubation with CsuA/B or CsuE anti-serum (1:100 dilution in 5% FCS/PBS, 4 °C overnight). After washing with PBS, CsuA/B or CsuE was detected with goat anti-rabbit secondary antibodies conjugated to Alexa488 or Alexa555 (1: 200 dilution; 1 h at RT). Nuclei were counterstained with DAPI (5 min, RT) and examined using a Leica SP8 inverted confocal laser system (Leica Microsystems) equipped with a HC PL APO 63×/1.40 oil immersion lens. Images were captured and processed using the LasX (Leica Microsystems) and ImageJ software^61^.

### Bacterial adherence to epithelial cells

For detection of bacterial adherence to epithelial cells, A549 lung carcinoma cells (3 × 10^4^) were seeded in 8-well chamber slides and incubated overnight at 37 °C with 5% CO_2_. The following day, cells were infected with selected strains of *A. baumannii* at an MOI of 1:100 for 4 h in serum free RPMI 1640 media. To prepare inoculum, *A. baumannii* strains were grown to stationary phase in LB liquid medium at 37 °C. Otherwise, the conditions of bacterial culture are described in results. At the end of the infection, non-adhered bacteria were removed by three times washing with PBS. Cells were kept in serum-free RPMI 1640 media, and bright-field images were captured with a fluorescence microscope (Nikon, Eclipse Ti). Images were processed using the NIS-Elements (Nikon) and ImageJ software^61^.

For CFU counts, the 3^rd^ and 4th dilutions of every well was processed for enumeration of bacterial CFU, following overnight incubation on LB agar plates.

For adherence to colon carcinoma epithelial cells, SW480 cell lines were kept in RPMI 1640 supplemented with 5% fatal bovine serum (FBS). Cells were seeded in 24-well cell culture plates (1 × 10^5^ cells/well), overnight at 37 °C and 5% CO_2_. A monolayer of SW480 cells was infected with bacterial suspension in PBS at a MOI of 1:100. Upon infection, the plate was incubated at 37 °C with 5% CO_2_ for 4 h. After completion of incubation, the monolayer was washed three times with PBS before the cells were detached by treatment with trypsin and then diluted in PBS. The 2^nd^ dilution of every well was processed for the enumeration of bacterial CFU, following overnight incubation on LB agar plates. The CFU numbers of each strain were used for the calculation of bacterial adherence as the percentage of bacterial cells associated with eukaryotic cells relative to the input. Each experiment was performed with three biological and three technical replicates.

### Invasion assay

For the invasion assay, bacterial growth conditions are indicated in figure legends and results. Bacteria were diluted in RPMI-1640 medium and subsequently seeded on confluent A549 cells grown in 96-well plates at a multiplicity of infection of 100 and incubated at in CO_2_ incubator for 4 h. After 4 h, the plates were centrifuged at 1500 *g* for 5 min. The supernatant was removed, and RPMI-1640 medium containing gentamicin at a final concentration of 100 µg/mL was added to the cells for 1 h to kill the remaining extracellular bacteria. Cells were gently washed twice with PBS and disrupted with 1% Triton X-100 (Sigma Chemical). The number of intracellular bacteria was determined by colony forming units (CFU) counts of viable bacteria. The relative invasion rate is defined as (invasion rate of mutant/invasion rate of wild type) *100 as shown previously^64^.

### MIC determination of colistin and carbencillin

The minimal inhibitory concentration of colistin and carbencillin was determined by the standard microbroth dilution method^65^. Bacterial inoculum equivalent to 0.5 McFarland (5 × 10^8^) was prepared and diluted 1:10 in Muller Hinton Broth to achieve the final inoculum of 5 × 10^7^ CFU/ml. Concentration range of the antibiotics to be tested was 0.125–64 µg using 96-well plates. The plates were incubated for 24 h at 37 °C, and the lowest concentration at which bacterial growth was completely inhibited was noted and declared the MIC.

### In vivo infection assay using a murine model of intraperitoneal or of intranasal infection

Bacterial strains were grown on LB agar plates overnight at 37 °C. From the overnight cultures, diluted samples were sub-cultured in LB broth until the logarithmic growth phase. An inoculum consisting of 10^7^ bacteria from the log phase culture was used to infect animals.

All animals were maintained and treated in accordance with the strict guidelines and permit issued by the Ethical Review Committee at the University of Health Sciences, Lahore, issued by the ethical and research committee through letter no. UHS/REG-19/ERC/1236. All steps were performed aseptically^66^.

Adult BALB/c mice were inoculated with the inoculum corresponding to 10^7^ bacteria intraperitoneally as described previously^66^ or intranasally. Animals were maintained under standard laboratory conditions with free access to food and water throughout the experimental period. In the first round of experiments, the killing time was recorded upon infection with wild-type and mutant strains. In subsequent experiments, mice were sacrificed after 20 h of infection. For the neutrophil count, 500 µl of the blood were withdrawn through a heart puncture and stored in vials containing 50 µl of 0.5 M EDTA. Mice were anesthesized with light ether vapours for blood samples collection. After blood collection, mice were euthanized with an overdose of ether vapours^67^. The relevant organs (such as lungs and spleens) were aseptically removed and used for quantitative bacteriology. Organs were homogenized in sterile saline using aerosol-proof homogenizers. Aliquots (100 μl) of 10-fold serial dilutions of the homogenates were cultured on LB agar plates to quantify the number of viable *A. baumannii* organisms in the respective organs. Total white blood cells and neutrophil count were measured by a haematology analyzer (Beckman Coulter DxH 900).

### Statistical analysis

Statistical significance of the difference was measured by non-parametric two tailed *t* test using Graph pad Prism 7. *p* values are shown by asterisk symbol. A single asterisk * indicates *p* ≤ 0.05, two asterisks ** indicate *p* ≤ 0.01, three asterisks *** indicate *P* < 0.001 and non-significant difference is shown by ns. The difference was compared to the relevant wild type strain otherwise indicated by horizontal bars above column bars.

### Reporting summary

Further information on research design is available in the Nature Research Reporting Summary linked to this article.

### Supplementary information

Supplementary Information

Reporting Summary

## Data Availability

All data supporting the findings of this study are included in the article and its supplementary files and figures. Additional data are available from the corresponding authors upon request.

## References

[1] Mea HJ, Yong PVC, Wong EH (2021). An overview of Acinetobacter baumannii pathogenesis: Motility, adherence and biofilm formation. Microbiol. Res..

[2] Harding CM, Hennon SW, Feldman MF (2018). Uncovering the mechanisms of Acinetobacter baumannii virulence. Nat. Rev. Microbiol..

[3] Ibrahim S, Al-Saryi N, Al-Kadmy IMS, Aziz SN (2021). Multidrug-resistant Acinetobacter baumannii as an emerging concern in hospitals. Mol. Biol. Rep..

[4] Ayoub Moubareck, C. & Hammoudi Halat, D. Insights into Acinetobacter baumannii: A review of microbiological, virulence, and resistance traits in a threatening nosocomial pathogen. *Antibiotics* **9**. 10.3390/antibiotics9030119 (2020). 10.3390/antibiotics9030119 PMC7148516 32178356

[5] Dijkshoorn L, Nemec A, Seifert H (2007). An increasing threat in hospitals: multidrug-resistant Acinetobacter baumannii. Nat. Rev. Microbiol..

[6] Eckardt, P. et al. Containment of a Carbapenem-resistant Acinetobacter baumanniii complex outbreak in a COVID-19 intensive care unit. Am. J. Infect. Control. 10.1016/j.ajic.2022.02.022 (2022). 10.1016/j.ajic.2022.02.022 PMC8881223 35227793

[7] Moradi N, Kazemi N, Ghaemi M, Mirzaei B (2021). Frequency and antimicrobial resistance pattern of bacterial isolates from patients with COVID-19 in two hospitals of Zanjan. Iran. J. Microbiol..

[8] Jeong, S. et al. Prevalence and Clinical impact of coinfection in patients with Coronavirus Disease 2019 in Korea. *Viruses* **14**. 10.3390/v14020446 (2022). 10.3390/v14020446 PMC8876760 35216039

[9] Aydemir O (2022). Secondary bacterial infections in patients with coronavirus disease 2019-associated pneumonia. Rev. Assoc. Med. Bras. (1992).

[10] Nebreda-Mayoral, T. et al. Bacterial/fungal infection in hospitalized patients with COVID-19 in a tertiary hospital in the Community of Castilla y León, Spain. *Enferm. Infecc. Microbiol. Clin. (Engl Ed)*. 10.1016/j.eimce.2022.02.002 (2022). 10.1016/j.eimce.2022.02.002 PMC8847094 35216948

[11] Choi CH, Lee JS, Lee YC, Park TI, Lee JC (2008). Acinetobacter baumannii invades epithelial cells and outer membrane protein A mediates interactions with epithelial cells. BMC Microbiol..

[12] Weber BS, Harding CM, Feldman MF (2015). Pathogenic Acinetobacter: from the cell surface to infinity and beyond. J. Bacteriol..

[13] Giannouli M (2013). Virulence-related traits of epidemic Acinetobacter baumannii strains belonging to the international clonal lineages I-III and to the emerging genotypes ST25 and ST78. BMC Infect. Dis..

[14] Bonomo RA, Szabo D (2006). Mechanisms of multidrug resistance in Acinetobacter species and Pseudomonas aeruginosa. Clin. Infect. Dis..

[15] Khazaal SS, Al-Saryi N, Ibrahim SA (2020). Immunomodulation by Acinetobacter baumannii of endotracheal tube biofilm in ventilator-associated pneumonia. Meta Gene.

[16] Tomaras AP, Dorsey CW, Edelmann RE, Actis LA (2003). Attachment to and biofilm formation on abiotic surfaces by Acinetobacter baumannii: involvement of a novel chaperone-usher pili assembly system. Microbiology.

[17] Eijkelkamp BA (2011). Adherence and motility characteristics of clinical Acinetobacter baumannii isolates. FEMS Microbiol. Lett..

[18] de Breij A (2009). CsuA/BABCDE-dependent pili are not involved in the adherence of Acinetobacter baumannii ATCC19606T to human airway epithelial cells and their inflammatory response. Res. Microbiol..

[19] Lee JC (2006). Adherence of Acinetobacter baumannii strains to human bronchial epithelial cells. Res. Microbiol..

[20] Harding, C. M. et al. Acinetobacter baumannii strain M2 produces type IV pili which play a role in natural transformation and twitching motility but not surface-associated motility. *mBio* **4**. 10.1128/mBio.00360-13 (2013). 10.1128/mBio.00360-13 PMC3735195 23919995

[21] Piepenbrink KH (2016). Structural diversity in the Type IV Pili of multidrug-resistant Acinetobacter. J. Biol. Chem..

[22] Ronish LA, Lillehoj E, Fields JK, Sundberg EJ, Piepenbrink KH (2019). The structure of PilA from Acinetobacter baumannii AB5075 suggests a mechanism for functional specialization in Acinetobacter type IV pili. J. Biol. Chem..

[23] Pakharukova N (2018). Structural basis for Acinetobacter baumannii biofilm formation. Proc. Natl Acad. Sci. USA.

[24] Pakharukova N (2015). Structural Insight into Archaic and alternative Chaperone-Usher pathways reveals a novel mechanism of Pilus Biogenesis. PLoS Pathog..

[25] Nuccio SP, Bäumler AJ (2007). Evolution of the chaperone/usher assembly pathway: fimbrial classification goes Greek. Microbiol. Mol. Biol. Rev..

[26] Choudhury D (1999). X-ray structure of the FimC-FimH chaperone-adhesin complex from uropathogenic Escherichia coli. Science.

[27] Sauer FG (1999). Structural basis of chaperone function and pilus biogenesis. Science.

[28] Pakharukova, N. et al. Archaic chaperone-usher pili self-secrete into superelastic zigzag springs. *Nature*. 10.1038/s41586-022-05095-0 (2022). 10.1038/s41586-022-05095-0 PMC9452303 35853476

[29] Romero, M. et al. Mushroom-shaped structures formed in Acinetobacter baumannii biofilms grown in a roller bioreactor are associated with quorum sensing-dependent Csu-pilus assembly. *Environ. Microbiol*. 10.1111/1462-2920.15985 (2022). 10.1111/1462-2920.15985 PMC9790458 35352448

[30] López-Martín, M., Dubern, J. F., Alexander, M. R. & Williams, P. AbaM Regulates Quorum sensing, biofilm formation, and virulence in Acinetobacter baumannii. *J. Bacteriol.* **203**. 10.1128/jb.00635-20 (2021) 10.1128/JB.00635-20 PMC8088503 33495249

[31] Lázaro-Díez M (2016). Acinetobacter baumannii and A. pittii clinical isolates lack adherence and cytotoxicity to lung epithelial cells in vitro. Microbes Infect..

[32] Chen C-L (2022). d-mannose-sensitive pilus of Acinetobacter baumannii is linked to biofilm formation and adherence onto respiratory tract epithelial cells. J. Microbiol. Immunol. Infect..

[33] Ellison CK (2022). Subcellular localization of type IV pili regulates bacterial multicellular development. Nat. Commun..

[34] Flannery A, Le Berre M, Pier GB, O’Gara JP, Kilcoyne M (2020). Glycomics microarrays reveal differential in situ presentation of the biofilm Polysaccharide Poly-N-acetylglucosamine on Acinetobacter baumannii and Staphylococcus aureus Cell Surfaces. Int. J. Mol. Sci..

[35] Karah N, Khalid F, Wai SN, Uhlin BE, Ahmad I (2020). Molecular epidemiology and antimicrobial resistance features of Acinetobacter baumannii clinical isolates from Pakistan. Ann. Clin. Microbiol. Antimicrob..

[36] Hospenthal MK, Costa TRD, Waksman G (2017). A comprehensive guide to pilus biogenesis in Gram-negative bacteria. Nat. Rev. Microbiol.

[37] Werneburg, G. T. & Thanassi, D. G. Pili Assembled by the Chaperone/Usher pathway in Escherichia coli and Salmonella. *EcoSal Plus* **8**. 10.1128/ecosalplus.ESP-0007-2017 (2018). 10.1128/ecosalplus.esp-0007-2017 PMC5940347 29536829

[38] Zeiner SA, Dwyer BE, Clegg S (2012). FimA, FimF, and FimH are necessary for assembly of type 1 fimbriae on Salmonella enterica serovar Typhimurium. Infect. Immun..

[39] Mannan T, Rafique MW, Bhatti MH, Matin A, Ahmad I (2020). Type 1 Fimbriae and motility play a pivotal role during interactions of Salmonella typhimurium with Acanthamoeba castellanii (T4 Genotype). Curr. Microbiol..

[40] Balázs, B. et al. Faecal carriage of Carbapenem-resistant Acinetobacter baumannii: Comparison to clinical isolates from the same period (2017–2019). *Pathogens* **11**. 10.3390/pathogens11091003 (2022). 10.3390/pathogens11091003 PMC9506371 36145435

[41] Dijkshoorn L (2005). Prevalence of Acinetobacter baumannii and other Acinetobacter spp. in faecal samples from non-hospitalised individuals. Clin. Microbiol. Infect..

[42] Ahmad I, Nygren E, Khalid F, Myint SL, Uhlin BE (2020). A Cyclic-di-GMP signalling network regulates biofilm formation and surface-associated motility of Acinetobacter baumannii 17978. Sci. Rep..

[43] Guo Q (2022). Elongation factor P modulates Acinetobacter baumannii physiology and virulence as a cyclic dimeric guanosine monophosphate effector. Proc. Natl Acad. Sci. USA.

[44] Clemmer KM, Bonomo RA, Rather PN (2011). Genetic analysis of surface motility in Acinetobacter baumannii. Microbiology.

[45] Jacobs AC (2014). AB5075, a highly virulent isolate of Acinetobacter baumannii, as a Model strain for the evaluation of pathogenesis and antimicrobial treatments. mBio.

[46] Ramezanalizadeh F, Owlia P, Rasooli I (2020). Type I pili, CsuA/B and FimA induce a protective immune response against Acinetobacter baumannii. Vaccine.

[47] Vijayakumar S (2016). Biofilm formation and motility depend on the nature of the Acinetobacter baumannii Clinical Isolates. Front Public Health.

[48] Moon, K. H., Weber, B. S. & Feldman, M. F. Subinhibitory Concentrations of Trimethoprim and Sulfamethoxazole Prevent Biofilm Formation by Acinetobacter baumannii through Inhibition of Csu Pilus Expression. *Antimicrob. Agents Chemother.* **61**. 10.1128/aac.00778-17 (2017). 10.1128/AAC.00778-17 PMC5571315 28674047

[49] Vesel, N. & Blokesch, M. Pilus Production in Acinetobacter baumannii Is Growth Phase Dependent and Essential for Natural Transformation. *J. Bacteriol.* **203**, 10.1128/jb.00034-00021 (2021). 10.1128/JB.00034-21 PMC8088505 33495250

[50] Monteiro C (2012). Hfq and Hfq-dependent small RNAs are major contributors to multicellular development in Salmonella enterica serovar Typhimurium. RNA Biol..

[51] Giraud C (2011). The PprA-PprB two-component system activates CupE, the first non-archetypal Pseudomonas aeruginosa chaperone-usher pathway system assembling fimbriae. Environ. Microbiol..

[52] Ruer S, Stender S, Filloux A, de Bentzmann S (2007). Assembly of fimbrial structures in Pseudomonas aeruginosa: functionality and specificity of chaperone-usher machineries. J. Bacteriol..

[53] Mikkelsen H, Ball G, Giraud C, Filloux A (2009). Expression of Pseudomonas aeruginosa CupD fimbrial genes is antagonistically controlled by RcsB and the EAL-containing PvrR response regulators. PLoS One.

[54] Blaschke U, Skiebe E, Wilharm G (2021). Novel genes required for surface-associated motility in Acinetobacter baumannii. Curr. Microbiol..

[55] Luo, Y. et al. A hierarchical cascade of second messengers regulates Pseudomonas aeruginosa surface behaviors. *mBio* **6**. 10.1128/mBio.02456-14 (2015). 10.1128/mBio.02456-14 PMC4324313 25626906

[56] Kuchma SL, O’Toole GA (2022). Surface-Induced cAMP signaling requires multiple features of the Pseudomonas aeruginosa Type IV Pili. J. Bacteriol..

[57] Webster SS (2022). Force-induced changes of PilY1 drive surface sensing by Pseudomonas aeruginosa. mBio.

[58] Fürste JP (1986). Molecular cloning of the plasmid RP4 primase region in a multi-host-range tacP expression vector. Gene.

[59] Tucker AT (2014). Defining gene-phenotype relationships in Acinetobacter baumannii through one-step chromosomal gene inactivation. mBio.

[60] Heydorn A (2000). Quantification of biofilm structures by the novel computer program COMSTAT. Microbiology.

[61] Schindelin J (2012). Fiji: an open-source platform for biological-image analysis. Nat. Methods.

[62] Pakharukova N (2018). Archaic and alternative chaperones preserve pilin folding energy by providing incomplete structural information. J. Biol. Chem..

[63] Ahmad I, Karah N, Nadeem A, Wai SN, Uhlin BE (2019). Analysis of colony phase variation switch in Acinetobacter baumannii clinical isolates. PLoS One.

[64] Ahmad I (2011). Complex c-di-GMP signaling networks mediate transition between virulence properties and biofilm formation in Salmonella enterica serovar Typhimurium. PLoS One.

[65] Ahsan, U. et al. Emergence of high colistin resistance in carbapenem resistant Acinetobacter baumannii in Pakistan and its potential management through immunomodulatory effect of an extract from Saussurea lappa. *Front. Pharmacol.* **13**. 10.3389/fphar.2022.986802 (2022). 10.3389/fphar.2022.986802 PMC9523213 36188613

[66] Harris G, KuoLee R, Xu HH, Chen W (2017). Mouse models of Acinetobacter baumannii infection. Curr. Protoc. Microbiol..

[67] Muhammad H (2023). Immunomodulatory effect of glabridin in ovalbumin-induced allergic asthma and its comparison with methylprednisolone in a preclinical rodent model. J. Cell Biochem..

