# Supplementary material for: Csu pili dependent biofilm formation and virulence of Acinetobacter baumannii
Source: NPJ Biofilms Microbiomes. 2023 Dec 14;9:101. doi: 10.1038/s41522-023-00465-6 (PMC10721868; doi:10.1038/s41522-023-00465-6)
Supplement: Supplementary file 1 — Supplementary Information [file 41522_2023_465_MOESM1_ESM.pdf]

Supplementary Information

Csu pili dependent biofilm formation and virulence of *Acinetobacter baumannii*

Irfan Ahmad[1,2,*], Aftab Nadeem[1], Fizza Mushtaq[1,2], Nikola Zlatkov[1], Muhammad Shahzad[4], Anton V. Zavialov[5], Sun Nyunt Wai [1,3], Bernt Eric Uhlin[1]

[1]Department of Molecular Biology and Umeå Centre for Microbial Research (UCMR), Umeå University, SE-90187 Umeå, Sweden

[2]Institute of Biomedical and Allied Health Sciences, University of Health Sciences, Lahore, Pakistan

[3]The Laboratory for Molecular Infection Medicine Sweden (MIMS), Umeå University, SE-90187 Umeå, Sweden

[4]Department of Pharmacology, University of Health Sciences, Lahore

[5]Department of Biochemistry, University of Turku; Tykistökatu 6A, 20520, Turku, Finland

*Correspondence:

Irfan Ahmad

irfan.ahmad@umu.se

Supplementary Figures 1-7

Supplementary Tables 1-4

Supplementary References

Supplementary Figure 1

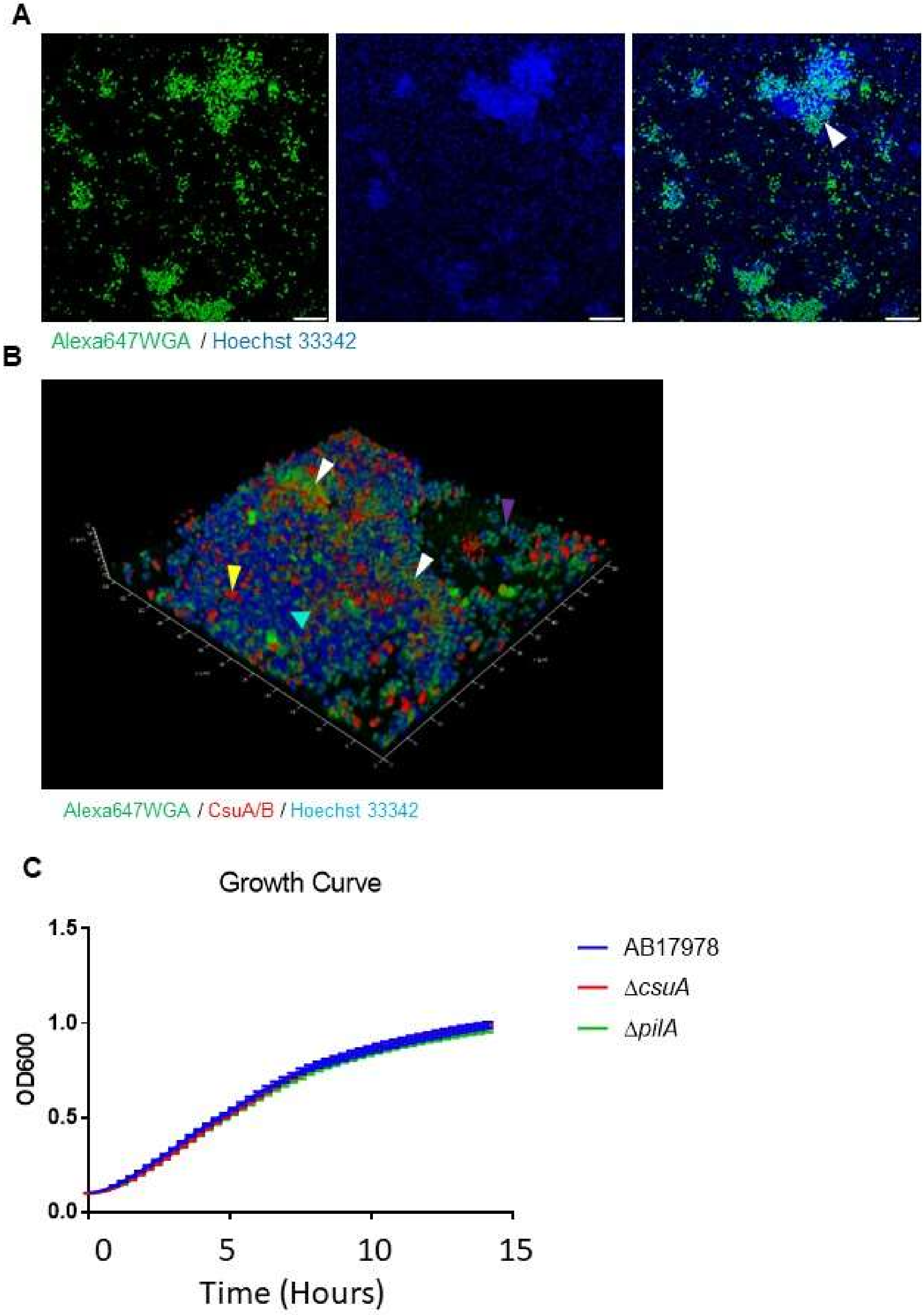

Supplementary Figure 1: A single section of biofilm patches formed on a glass surface, followed by labelling with Hoechst 33342 (blue) and Alexa647-WGA (green). Scale bars = 20 μm. (B) Three-dimensional confocal laser microscopic visualization of CsuA/B expression (red), PNAG expression (green) and CsuA-PNAG co-expression within a typical patch of biofilm formed on a glass surface by *A. baumannii* 17978. The arrowheads in white indicate co-localization of Alexa647-WGA (green) and CsuA/B (red), yellow indicates localization of CsuAB (red), cyan indicates Hoechst 33342 (blue) stained cells, and purple shows Alexa647-WGA (green) stained structures. (C) The growth curves of *A. baumannii* 17978 and mutant strains grown in a microtitter plate in LB medium at 37ºC using the built-in temperature control mode of the Spark multimode plate reader (Tecan). Y- axis represents the optical density (OD_{600}) measured with an interval of 20 minutes for 16 hours. The experiment was done in triplicate, and the curves were drawn using the mean OD_{600} values.

Supplementary Figure 2

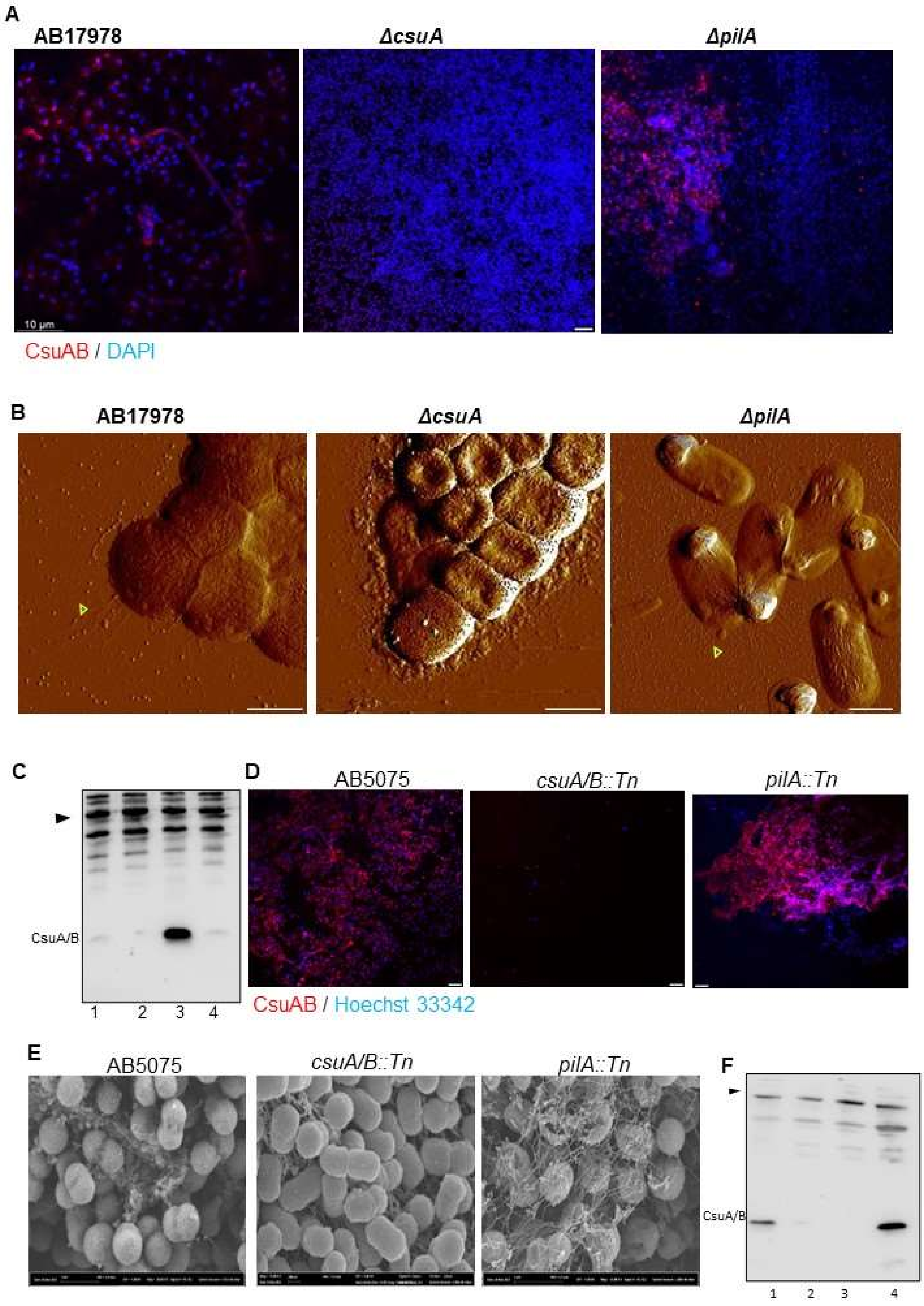

Supplementary Figure 2: (A) Confocal laser microscopic imaging of biofilms formed by *A. baumannii* 17978, *ΔcsuA* and *ΔpilA* mutants. Red: CsuA/B immunostained with CsuA/B specific antisera, Blue DAPI. (B) Atomic force microscopic imaging of the samples prepared from the pellicles of *A. baumannii* 17978 wild-type and mutant strains. Scale bars = 1 μm. (C) Western blot analysis of CsuA/B subunit in pellicles formed by *A. baumannii* 17978 and its mutant strains1- AB17978-vector control, 2- Δ*csuA*-vector control, 3- Δ*pilA*-Vector control, 4- Δ*csuA*-pCsuA. The arrowhead indicates equal distribution of proteins. (D) Confocal laser microscopy images of a pellicle sample labelled with CsuA/B antiserum (red) and Hoechst 33342 nuclear stain (blue) illustrating the expression of CsuA/B subunits in the pellicles of *A. baumannii* AB5075 wild-type and transposon mutants. The size bars in the images correspond to 10μm. (E) Scanning electron microscopic images of pellicle formed by *A. baumannii* AB5075 wild type and mutant strains. (F) A representative Western blot image to show expression of the CsuA/B in *A. baumannii* AB5075 wild type strain cultivated after growth at different growth conditions. 1- LB broth after 48 hours at 37ºC. 2- LB plates after 48 hours at 37ºC, 3- LB agar plate lacking NaCl ,4- pellicle formed within LB broth. The arrow-head indicates a nonspecific band as a loading control.

Supplementary Figure 3

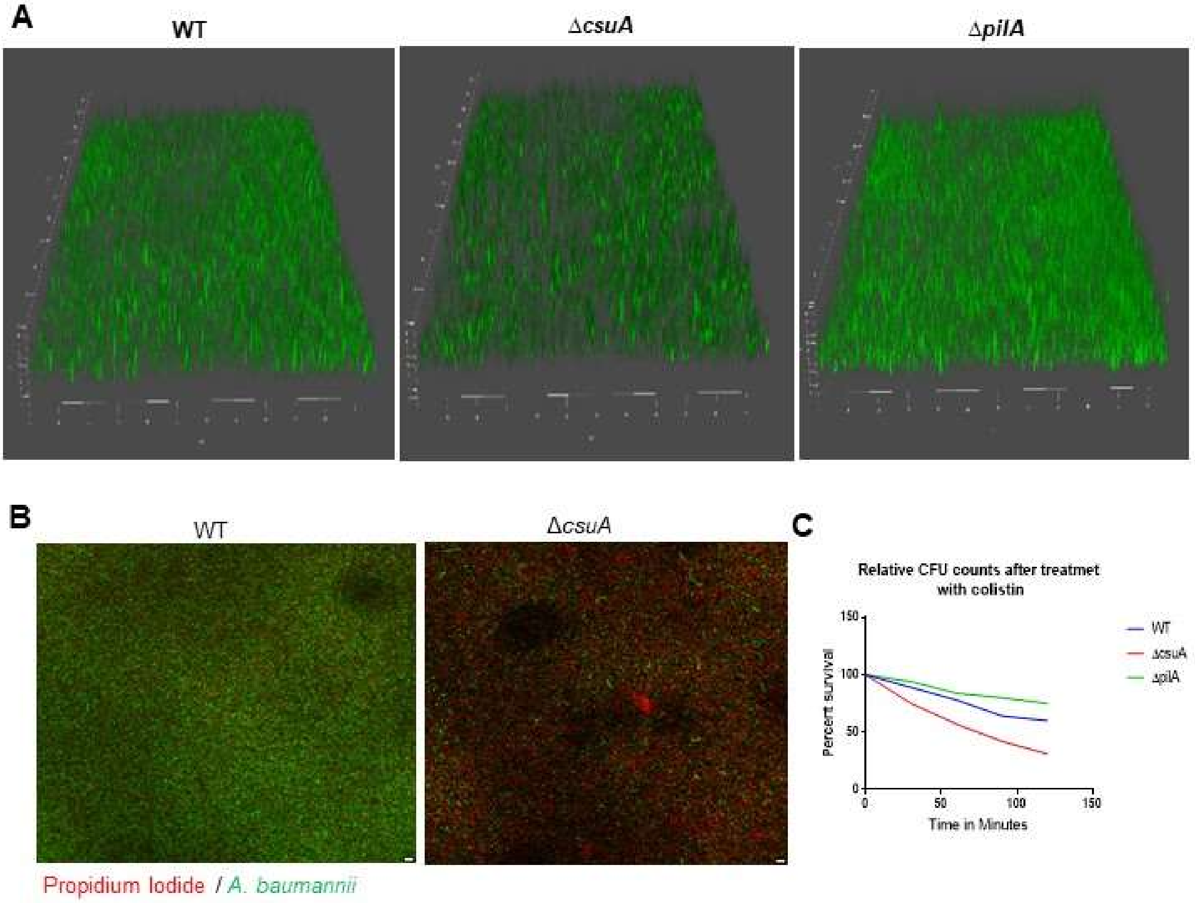

Supplementary Figure 3: (A) Live cell confocal laser microscopic images of biofilms formed by *A. baumannii* 17978 wild-type and mutant strains expressing green fluorescent protein within plastic cells after 24 hours of incubation at 30ºC. (B) Live cell images of biofilms in the plastic cells formed by wild-type and *ΔcsuA* mutant *A. baumannii* 17978 that were treated with 5 µg/ml of colistin for 2 hours. Cells were stained with Propidium Iodide (PI) to visualize dead bacteria within biofilms. The size bars in the images correspond to 5 µm. (C) Comparison of the percent survival of wild-type and mutant strains after treatment of biofilms with colistin, as measured through CFU counts of bacteria on agar plates.

Supplementary Figure 4

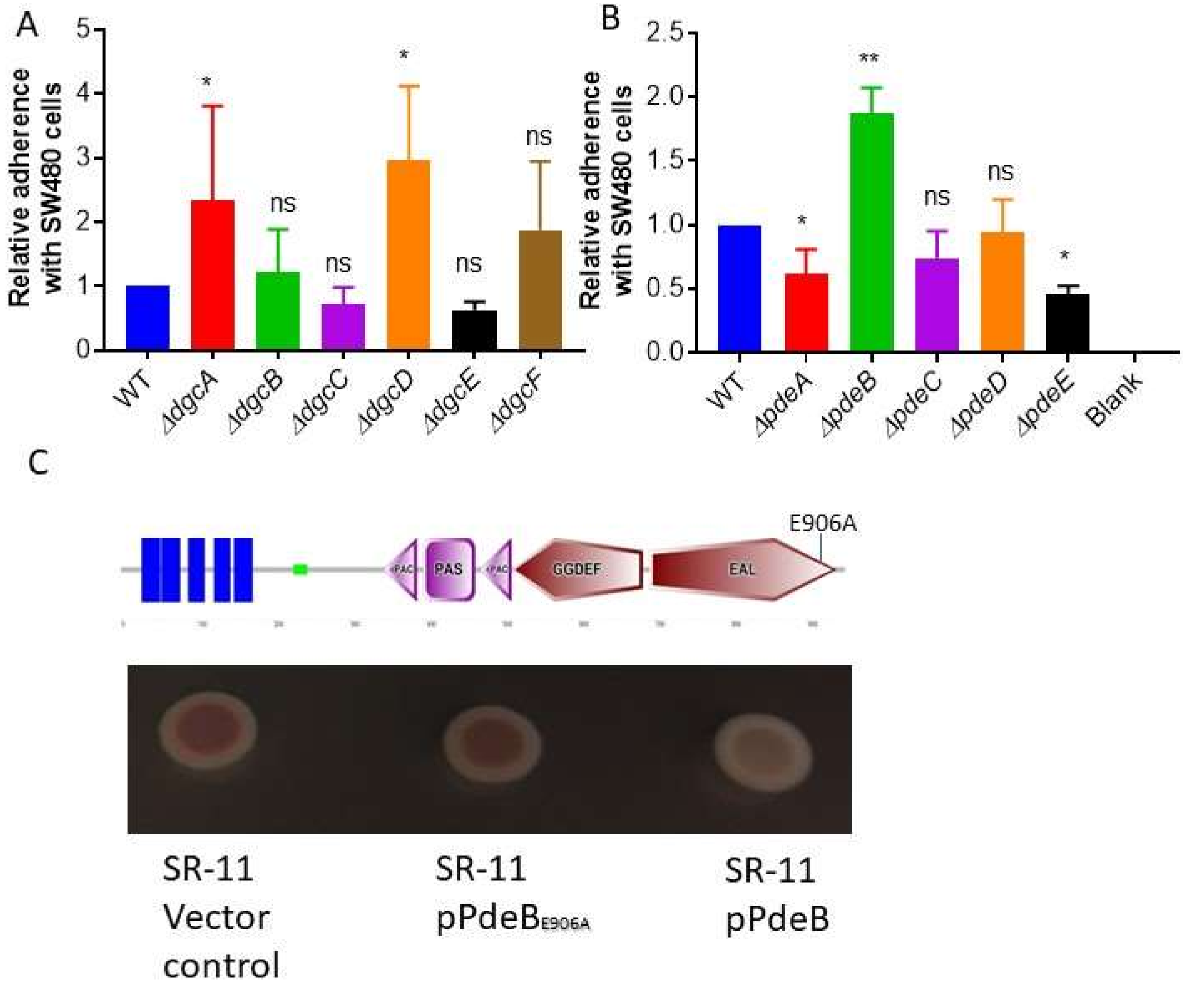

Supplementary Figure 4: Bar chart diagrams illustrating the screening of the individual mutants of 11 GGDEF/EAL proteins of *A. baumannii* 17978 for adherence to epithelial cells A549. Adherence of GGDEF mutants (A) and GGDEF-EAL and EAL mutants (B) to epithelial cells A549. Bars show $\pm$ standard deviation between the experiments. Statistical significance is indicated by *p* value that was measured by non-parametric two tailed *t* test. A single asterisk * indicates $p \leq 0{,}05$, two asterisks ** indicate $p \leq 0{,}01$ and “ns” indicates non-significant difference as compared to wild type. (C) Upper panel: Domain architecture of PdeB, alias AIS_2337, highlighting the position of glutamic acid 906, mutated to create a

catalytically inactive variant of PdeB. Lower Panel: Image of the Congo red agar plate containing spots of *S. typhimurium* SR-11 grown at 30ºC for 24 hours to illustrate the effect of E906A mutation on the phosphodiesterase activity of PdeB. Wild type PdeB inhibits the formation of the rdar morphotype, whereas $PdeB_{E906A}$ does not suppress rdar morphotype formation.

Supplementary Figure 5

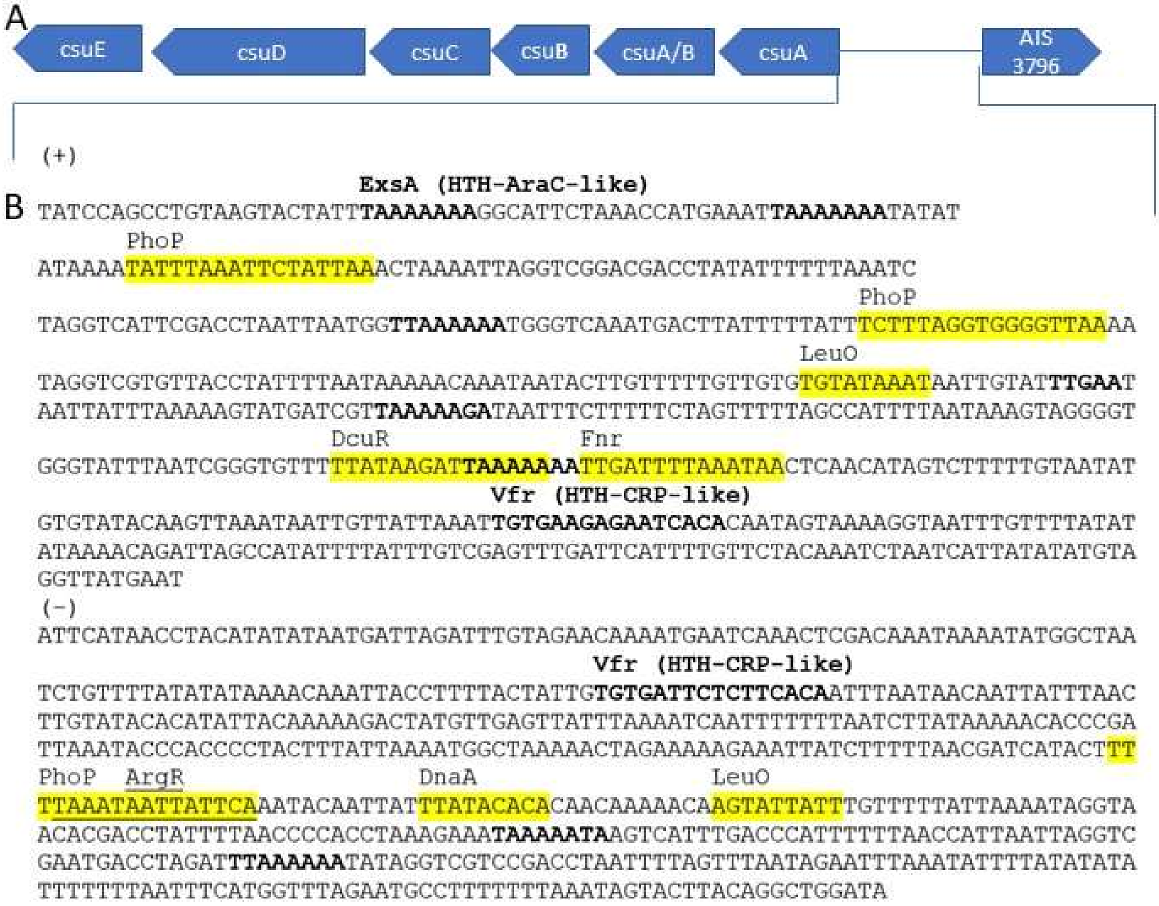

Supplementary Figure 5: (A) Organisation of the *csuABCDE* operon along with the promoter region (B) DNA sequence of the promoter region of *csu* operon highlighting putative binding sites for multiple transcriptional regulators.

Supplementary Figure 6

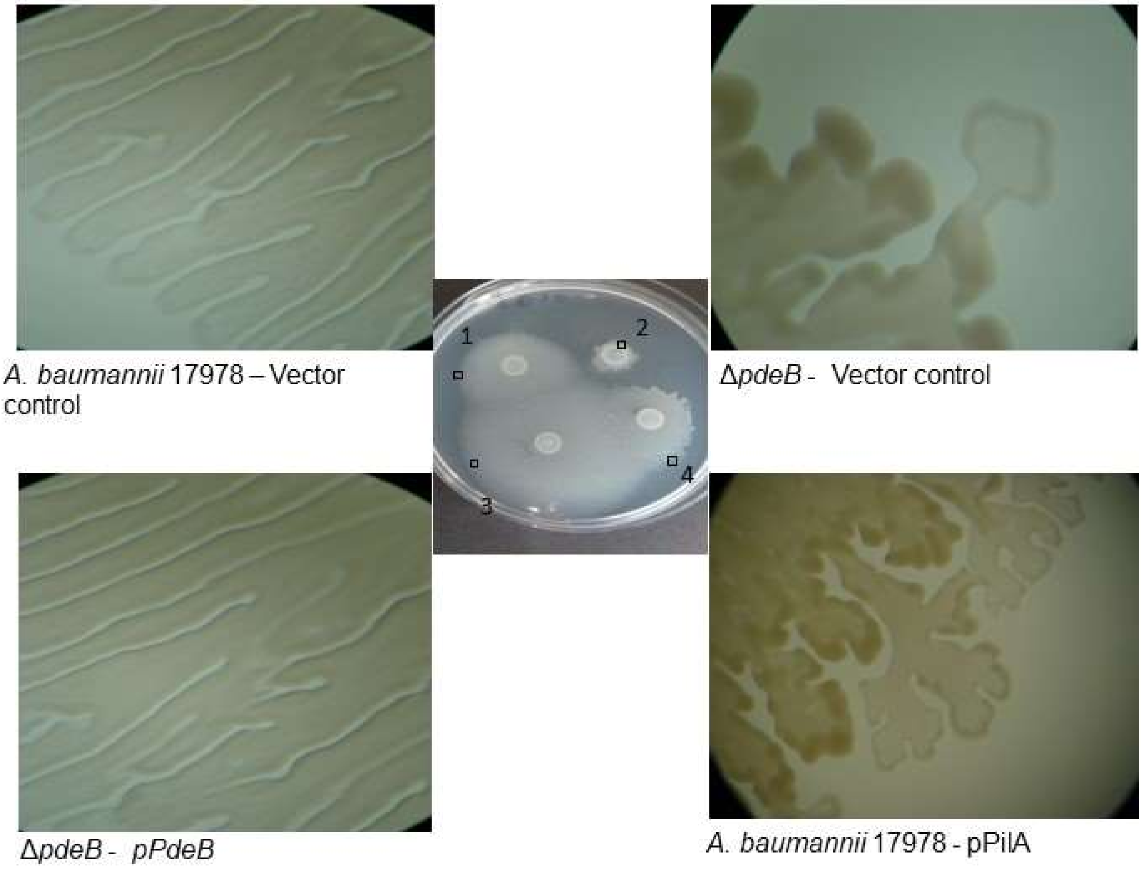

Supplementary Figure 6: Stereomicroscopic images of surface-associated motility exhibited by *A. baumannii* 17978 wild-type- vector control (1), Δ*pdeB*- vector control (2) Δ*pdeB* trans complemented strain from the plasmid pPdeB (3) and wildtype with over production of PilA from plasmid pMBB67EH (4).

Supplementary Figure 7: Full unedited images for Western blots

Unedited gel for Figure 1A

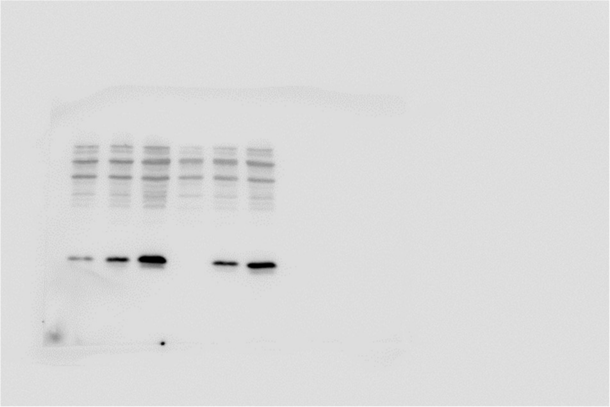

Unedited gel for Figure 2D

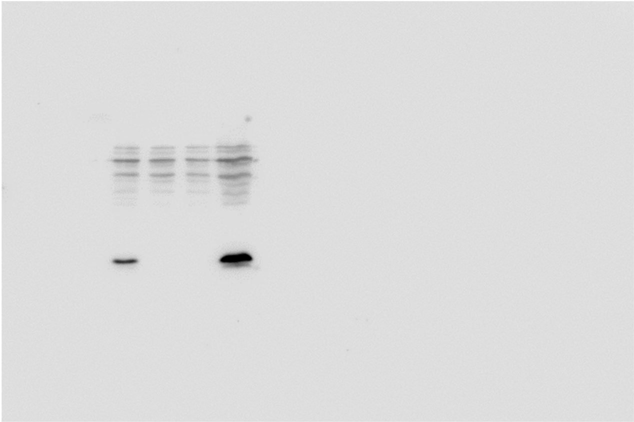

Unedited gel for Figure 3B

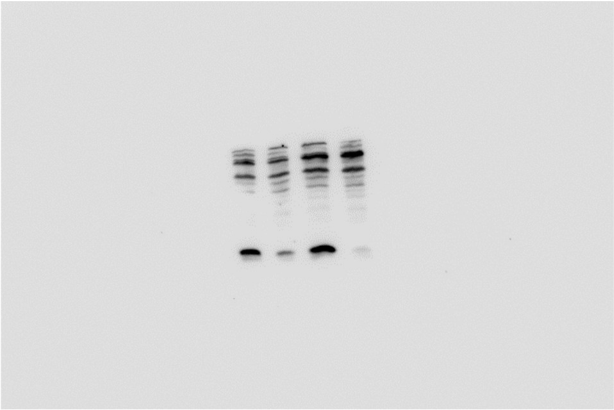

Unedited gel for Figure 5E

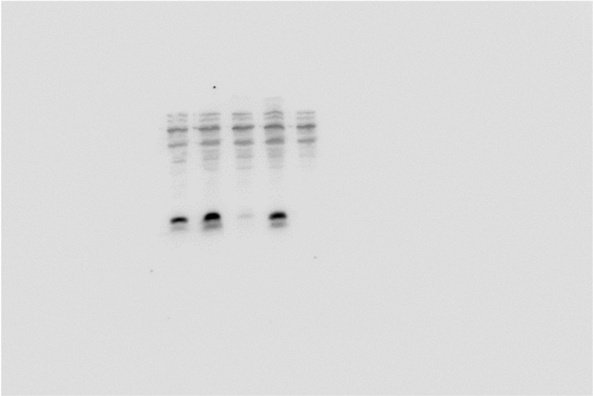

Supplementary Tables

Supplementary Table 1: Minimum inhibitory concentration of Colistin and carbenicillin in *A. baumannii* isolates

**Isolate**	**MIC of colistin**	**MIC of Carbenicillin**
AB17978	0,4 ug/ml	<8 ug/ml
AB17978 Δ*csuA*	0,4ug/ml	<8 ug/ml
AB17978 Δ*csuC*	0,4 ug/ml	<8 ug/ml
AB17978 Δ*pilA*	0,4 ug/ml	<8 ug/ml
AB5075	0,8 ug/ml	>64 ug/ml
AB5075 *csuA/B::Tn*	0,8 ug/ml	>64 ug/ml
AB5075 *pilA::Tn*	0,8 ug/ml	>64 ug/ml
AB5075 *pdeB::Tn*	0,8 ug/ml	>64 ug/ml

Supplementary Table 2: *Acinetobacter baumannii* strains used in the study

**Strain ID**	**Genotype**	**Reference / Source**
AB17978	Wild type	ATCC17978
AB17978 Δ*csuA*	Δ*csuA:*Km	This study
AB17978 Δ*csuC*	Δ*csuC:*Km	This study
AB17978 Δ*pilA*	Δ*pilA:*Km	This study
AB17978 Δ*dgcA*	Δ*AIS_0751:* Km	This study
AB17978 Δ*dgcF*	Δ*AIS_3296*: Km	[1]
AB17978 Δ*pdeA*	Δ*AIS_1949:* Km	[1]
AB17978 Δ*dgcB*	Δ*AIS_1067*: Km	This study
AB17978 Δ*pdeC*	Δ*AIS_1254*: Km	This study
AB17978 Δ*dgcD*	Δ*AIS_2506*: Km	This study
AB17978 Δ*pdeE*	Δ*AIS_2422*: Km	This study
AB17978 Δ*pdeD*	Δ*AIS_0546*: Km	This study
AB17978 Δ*dgcE*	Δ*AIS_2986*: Km	This study
AB17978 Δ*dgcC*	Δ*AIS_1695*: Km	This study
AB17978 Δ*pdeB*	Δ*AIS_2337*: Km	This study
AB5075	Wild type	[2]
AB5075 *csuA/B::Tn*	*ABUW_1487::Tn10-ATS*	[2]
AB5075 *pilA::Tn*	*ABUW_0304::Tn10*-ATS	[2]
AB5075 *pdeB::Tn*	*ABUW_*1221*::Tn10*-ATS	[2]
Ab-Pak-Pesh-22	Clinical isolate, bla_{OXA-68}, *ampC-79*, Sequence type 23	[3,4]
Ab-Pak-Pesh-37	Clinical isolate, $bla_{OXA-371}$, *ampC-3*, Sequence type 1106	[3,4]
Ab-Pak-Lah-14	Clinical isolate, $bla_{OXA-371}$, *ampC-8*, Sequence type 1	[3,4]

Supplementary Table 3: Plasmids used in the study

Plasmid	Description	Reference
pMMB67EH	Vector plasmid for cloning	[5]
pAT02	RecAB cloned in pMBB67EH	Tucker, et al. [6]
pCsuA	CsuAx6His cloned in pMBB67EH	This study
p3296	AIS_3296x6His cloned in pMBB67EH	[1]
pPdeB	AIS_2337x6His cloned in pMBB67EH	[1]
pPilA	PilAx6His cloned in pMBB67EH	This study

Supplementary Table 4: Oligonucleotide primers used in the study

Primer	5`-3´ Sequence
Primers used to delete genes in *A. baumannii 17978*	
A1S_0751_KO_Forward	ATGATTGGTAGTTTTTGGGCCGAATTTGGTTATTGGGTTACTTCAGAGCTTCAAAATGCCTTATTGTTGCTGCCCATTATTTTAAACATCCCCCCTCCGGAATTGCCAGCTGGG
A1S_0751_KO_Reverse	TTACAAAGAAATAAAAACTCTATTACGCCCTTGGCGCTTTGCTTGATAGAGCGCATCGTCTGCTTCTTTAAACAAACTTTCAATAGAACGGTTCAGAAGAACTCGTCAAG
A1S_1695_ko_f	ATGAAGTTGCAAGGTTCCAATATATTGGGACAGGAACAAATAGATTTATTAACCACACGCGGATTAAATTTCGTATGGTTTCCAAAGCAACTTGAACCGGAATTGCCAGCTGGG
A1S_1695_ko_r	TTAAGTTAGATCTACAATTTGTTCTTCATTCAAAGCAATTTGATATTGATTGCGGCCATTTGTTTTTGCCTGATAGAGCGCATGATCAGCATTCAGAAGAACTCGTCAAG
A1S_2506_Ko_Forward	ATGGAAACTTTAGATTCTTCAATTTTTGACCTCACCCCAATTCCAATGTGGATTGAAGACTTTAGTGAAGTTAAGCAGTTATTTGACTTATGGAGACCGGAATTGCCAGCTGGG
A1S_2506_Ko_Reverse	TTATGGTAGAAGTAATTTCTTGATAATAACTTTTTTCTTTGAATACATATGATGATCTGCGCGTTTTAGCATATCTTCAACTTGTTCATTTTTCAGAAGAACTCGTCAAG
A1S_2986_Ko_F	ATGGAAACTTATGCAAAACAAAATTTGCAATTACTTAGCCATACTCTTCTTGAGCGTATTCAACCTGCCGTTGTGTTTAACGATAAGATCACGATCCCGGAATTGCCAGCTGGG
A1S_2986_Ko_R	TTAATTCTCTGGTTTATAAATAAACCAATGTTGCTCAGAAGTCTTAGCCTTATACATCGCTTGATCTGCTTGCATAATAAAATCTTCTGGATTCAGAAGAACTCGTCAAG

A1S_0546_ko_F	ATGGGTCATGTTGATTACGATAGCACATTGATCGTCGGCTCTTTTATTGCGGCTGGCGCTATTTGCTATATTGTGATTTCCATGGAGCAGTTAATACCGGAATTGCCAGCTGGG
A1S_0546_ko_R	TTAAACAAAATGAGCACCTTGCGACTGAATCAGTTGCCCTACATTTACAGGCATACCCAACAAATAACCTTGGAACTGCTGACAGCCCAAGTTCAGAAGAACTCGTCAAG
A1S_2337_ko_F1	ATGTCTGGCCTTCAGGAAGAATATTTACAGCATCAAACAAAAATTGAGCAGCTTCGCCTCTCAATTCAATATATCCGCTGGTATCTTGTTAGCCTACCGGAATTGCCAGCTGGG
A1S_2337_ko_R	TCAAATATGCGTATTTGTTTGTAGATATTGAAGAATCTGATTTGGATGAAGTGGACGGCTAAAGAAAAAGCCTTGTAAAAAATCACATTGCTTCAGAAGAACTCGTCAAG
A1S_2422_KO_F	TTTCTATATTACGCTGGTTCGGGCACTGTCATACAGCCGCCTTTTACAGACGGAACAACGTCTTTTGGCTGAGCTTGATACTATTCATACGCATACCCGGAATTGCCAGCTGGG
A1S_2422_KO_R	CCATGTTGGCGTAAGCGAGCAATGTGCTGTTGAGCAATTGGGAGATAAGGCTGAAGAGCTTGTTCTGAAAATTGCAAAATCAGTGGGCTTTTTCAGAAGAACTCGTCAAG
A1S_1067_KO_f	GTGGCGAATAGGGGAAATGTACATCAATTACTCAAATCTAAAGAAGAAATCGAGTATTTGGTCACTCTACATACCCATCGTTATGCCAATATGCCTTTGCCCCCGGAATTGCCAGCTGGG
A1S_1067_KO_R	TTAGGCAATTTCAGCGACTTGTAATGGCTGATAATAAATTTGATTACGGCCAAGCTGTTTTGCCCGGTATAAAGCCTGATCTGCCGCATGAATAAGCCTATTTCAGAAGAACTCGTCAAG
A1S_1254_KO_f	ATGGATATCTGTTTCACACTAATTTCCATATTTAGCTTTTTTCTTATTTATATAAACCGTATTTCGGCAGGGATACTTTTATCACAAGCGGTTCTACTGGTTCCGGAATTGCCAGCTGGG
A1S_1254_KO_R	TTATTCTTTATCGATCTTGATGGGCAATTGCTTGATAAACTCATTTAAAGACATTGGACAGCCCCATAAATAGCCCTGAAACTCGGTACAACCATTACTTTTTCAGAAGAACTCGTCAAG
pilA-ko-f	ATGAATGCACAAAAAGGTTTTACATTAATTGAACTCATGATCGTGGTTGCCATTATCGGTATTTTGGCAGCAATTGCGATTCCGCAATATCAGAAATACACTGTGTAGGCTGGAGCTGCTTC
pilA-ko-r	TTAAATTATTGTACAGCCTTTTGGAGCAATTACTGAAGAAATATCTGTTGTTCCTTTAGTAATAGAACATGTCCAGCCACCCACATTTGCATTTACTCCAGCATATGAATATCCTCCTTAGT
CsuA_Ko_F	ATGATATTCAATCGTGGTTCGGCATTTATAATTTCTTATTTTTTAATTTCTTTAGTAAATGCGGGTGAAATTGGAGCTAAATTAACTAGTCAAATTGAATCCGGAATTGCCAGCTGGG
CsuA_KO_R	TTAAAACTCAATCGTAATTGGTACTATATCTTTATATTCACCCTTAGATATACGACTACCATCATGGGTTGCTTGACCAAAAATATCGATATTCTTTTTATTCAGAAGAACTCGTCAAG

CsuC_ko_F	ATGAACAATTCTGCATTTATTAAAAATGGCATTTTAAAATCTTTTTTATTTGCAAGTACATTATCACTTGTTACACCTGTGATGGCACAAGCAACTTTTCCCGGAATTGCCAGCTGGG
CsuC_ko_R	TCATGATGGATCCTCCATTTTGGTGATTTCGATCAATTCTTGTTTAATACCAGAACTGTCCACACCATAAATTTTAGATGTTTTTGACAACTCATTCAGAAGAACTCGTCAAG
Primers used to confirm deletion of genes	
CsuA_Conrol_FOrward	GGCGATTATAAAGATACTC
CsuA_Conrol_Reverse	GAATTGAGCTGAGGATCT
CsuC_control_F	CCGTCTGGCTAAATTTTGA
CsuC_Control_R	CCACCCGACAATAAAATCA
A1S_0751_Control_F	TCGTATGGAGTAATGGCACTCAT
A1S_0751_Control_R	GTCGCACCAGTAATTGTGCAAAC
A1S_1695_control_F	AGACCATCCATTATGCGGCGTAA
A1S_1695_Control_R	GGATGTTGTTCCTCCATTTGCG
A1S_2506_control_Forward	CGACCACCCTATTAACCGTTAA
A1S_2506_control_Reverse	GGTACTCGTCGTTCTATGGGTAA
A1S_2986_Control_F	TTGGCTAAGAACGCGGAGTAA
A1S_2986_Control_R	GCACAGCAATGCAATAAAGG
A1S_0546-	CAAGCGAACAAAATCGTAGAC

_control_F	
A1S_0546-_control_R	GAGTTTAGAAGTGTCAATGTGG
A1S_2337_control_F	CGTACTGAAACTGCACCTGTTGTAGC
A1S_2337_control_R	GCGGCCTGTTTTAAAGATTGATCGTC
A1S_2422_control_F	GCCGCAGCTTCCAATATTAC
A1S_2422_control_R	GCTGCTGTAATATTGAATCGC
A1S_1067_control_f	GGAGCAGGGTAATAGAGTTCAA
A1S_1067_control_r	GGTTCTGCTGTACCTCAATTAG
A1S_1254_CONTROL_F	CGCAATAAGGTATAAGCTGAG
A1S_1254_CONTROL_R	ACTGCGCTTCAGGTTTCGTCAT
pilA-control-f	GCAATACCAGAAAGCTGTAGTTAC
pilA-control-rev	CTTAACCCTGCTGCAAAGGCA
Inside_Kan_Forw	CTGCTTGCCGAATATCATGG
Inside_Kan_Rev	CTCGTCCTGCAGTTCATTC
Primers used for cloning	
pilA-clon-f	CGTATCTAGAGGGAAAAAGGCTATGAATGCAC

pilA-clon-r	CGTCGAAGCTTTTAATGGTGATGGTGATGGTGAATTATTGTACAG CCTTTTGGAGC
Primers used for site directed mutagenesis	
pdeB_AGVE_F	GCTAGTTGCAGCAGGTGTGGAAAC
pdeB_AGVE_Rev	GTTTCCACACCTGCTGCAACTAGC

Supplementary References:

1 Ahmad, I., Nygren, E., Khalid, F., Myint, S. L. & Uhlin, B. E. A Cyclic-di-GMP signalling network regulates biofilm formation and surface associated motility of Acinetobacter baumannii 17978. *Sci Rep* **10**, 1991 (2020). https://doi.org:10.1038/s41598-020-58522-5

2 Gallagher, L. A. *et al.* Resources for Genetic and Genomic Analysis of Emerging Pathogen Acinetobacter baumannii. *J Bacteriol* **197**, 2027-2035 (2015). https://doi.org:10.1128/jb.00131-15

3 Ahmad, I., Karah, N., Nadeem, A., Wai, S. N. & Uhlin, B. E. Analysis of colony phase variation switch in Acinetobacter baumannii clinical isolates. *PLoS One* **14**, e0210082 (2019). https://doi.org:10.1371/journal.pone.0210082

4 Karah, N., Khalid, F., Wai, S. N., Uhlin, B. E. & Ahmad, I. Molecular epidemiology and antimicrobial resistance features of Acinetobacter baumannii clinical isolates from Pakistan. *Ann Clin Microbiol Antimicrob* **19**, 2 (2020). https://doi.org:10.1186/s12941-019-0344-7

5 Fürste, J. P. *et al.* Molecular cloning of the plasmid RP4 primase region in a multi-host-range tacP expression vector. *Gene* **48**, 119-131 (1986). https://doi.org:10.1016/0378-1119(86)90358-6

6 Tucker, A. T. *et al.* Defining gene-phenotype relationships in Acinetobacter baumannii through one-step chromosomal gene inactivation. *mBio* **5**, e01313-01314 (2014). https://doi.org:10.1128/mBio.01313-14
