# Supplementary material for: Csu pili dependent biofilm formation and virulence of Acinetobacter baumannii
Source: NPJ Biofilms Microbiomes. 2023 Dec 14;9:101. doi: 10.1038/s41522-023-00465-6 (PMC10721868; doi:10.1038/s41522-023-00465-6)
Supplement: Supplementary file 2 — Reporting Summary [file 41522_2023_465_MOESM2_ESM.pdf]

nature portfolio

Corresponding author(s): Irfan Ahmad
Last updated by author(s): Sep 1, 2023

Reporting Summary

Nature Portfolio wishes to improve the reproducibility of the work that we publish. This form provides structure for consistency and transparency in reporting. For further information on Nature Portfolio policies, see our Editorial Policies and the Editorial Policy Checklist.

Statistics

For all statistical analyses, confirm that the following items are present in the figure legend, table legend, main text, or Methods section.

n/a	Confirmed	
☐	☒	The exact sample size (*n*) for each experimental group/condition, given as a discrete number and unit of measurement
☐	☒	A statement on whether measurements were taken from distinct samples or whether the same sample was measured repeatedly
☐	☒	The statistical test(s) used AND whether they are one- or two-sided<br>*Only common tests should be described solely by name; describe more complex techniques in the Methods section.*
☒	☐	A description of all covariates tested
☒	☐	A description of any assumptions or corrections, such as tests of normality and adjustment for multiple comparisons
☐	☒	A full description of the statistical parameters including central tendency (e.g. means) or other basic estimates (e.g. regression coefficient) AND variation (e.g. standard deviation) or associated estimates of uncertainty (e.g. confidence intervals)
☒	☐	For null hypothesis testing, the test statistic (e.g. *F*, *t*, *r*) with confidence intervals, effect sizes, degrees of freedom and *P* value noted<br>*Give P values as exact values whenever suitable.*
☒	☐	For Bayesian analysis, information on the choice of priors and Markov chain Monte Carlo settings
☒	☐	For hierarchical and complex designs, identification of the appropriate level for tests and full reporting of outcomes
☒	☐	Estimates of effect sizes (e.g. Cohen's *d*, Pearson's *r*), indicating how they were calculated

Our web collection on statistics for biologists contains articles on many of the points above.

Software and code

Policy information about availability of computer code

Data collection	Leica SP8 inverted confocal system (Leica Microsystems) equipped with a HC PL APO 63x/1.40 oil immersion lens.<br>Spark® multimode microplate reader (Tecan).
Data analysis	GraphPad Prism version 7.05 (GraphPad Software) and Microsoft Excel for Mac Version 16.16.13<br>Analysis of images: ImageJ-FIJI distribution, Version 1.0 (National Institute of Health, USA), and LasX (Leica Microsystems).<br>The detailed information for data analysis is described in the Methods section.

For manuscripts utilizing custom algorithms or software that are central to the research but not yet described in published literature, software must be made available to editors and reviewers. We strongly encourage code deposition in a community repository (e.g. GitHub). See the Nature Portfolio guidelines for submitting code & software for further information.

Data

Policy information about availability of data

All manuscripts must include a data availability statement. This statement should provide the following information, where applicable:

- Accession codes, unique identifiers, or web links for publicly available datasets
- A description of any restrictions on data availability
- For clinical datasets or third party data, please ensure that the statement adheres to our policy

All data supporting the findings of this study are available within the paper and its Supplementary Information.

Research involving human participants, their data, or biological material

Policy information about studies with human participants or human data. See also policy information about sex, gender (identity/presentation), and sexual orientation and race, ethnicity and racism.

Reporting on sex and gender	Research does not include human participants, their data or biological material
Reporting on race, ethnicity, or other socially relevant groupings	Research does not include human participants, their data or biological material
Population characteristics	Research does not include human participants, their data or biological material
Recruitment	Research does not include human participants, their data or biological material
Ethics oversight	Research does not include human participants, their data or biological material

Note that full information on the approval of the study protocol must also be provided in the manuscript.

Field-specific reporting

Please select the one below that is the best fit for your research. If you are not sure, read the appropriate sections before making your selection.

☒ Life sciences ☐ Behavioural & social sciences ☐ Ecological, evolutionary & environmental sciences

For a reference copy of the document with all sections, see nature.com/documents/nr-reporting-summary-flat.pdf

Life sciences study design

All studies must disclose on these points even when the disclosure is negative.

Sample size	It was not a population based study. Therefore sample size was not calculated
Data exclusions	No data were excluded
Replication	For statistical analysis, every experiment was repeated at least three times
Randomization	Randomization was not relevant to the current study.
Blinding	The experiments were not blinded but repeated at least twice. The experiments were performed by independent researchers.

Reporting for specific materials, systems and methods

We require information from authors about some types of materials, experimental systems and methods used in many studies. Here, indicate whether each material, system or method listed is relevant to your study. If you are not sure if a list item applies to your research, read the appropriate section before selecting a response.

Materials & experimental systems

n/a	Involved in the study	
☐	☒	Antibodies
☐	☒	Eukaryotic cell lines
☒	☐	Palaeontology and archaeology
☐	☒	Animals and other organisms
☒	☐	Clinical data
☒	☐	Dual use research of concern
☒	☐	Plants

Methods

n/a	Involved in the study	
☒	☐	ChIP-seq
☒	☐	Flow cytometry
☒	☐	MRI-based neuroimaging

Antibodies

Antibodies used	CsuA/B rabbit polyclonal antibodies (custom produced by Innovagen AB), Goat anti-rabbit-HRP (#AS09602, WB: 1:5000) were purchased from Agrisera
Validation	Detailed construct and validation of primary antibody was performed in the study published previously DOI:https://doi.org/10.1074/jbc.RA118.004170

Eukaryotic cell lines

Policy information about cell lines and Sex and Gender in Research

Cell line source(s)	A495 lung epithelial cells, SW480 human rectal carcinoma cells
Authentication	cells were not athenticated
Mycoplasma contamination	All cell lines were tested for Mycoplasma contamination using PCR.
Commonly misidentified lines (See ICLAC register)	Commonly misidentified cell lines were not involved in the study

Animals and other research organisms

Policy information about studies involving animals; ARRIVE guidelines recommended for reporting animal research, and Sex and Gender in Research

Laboratory animals	Adult BALB/c mice were used in the study.
Wild animals	Study did not involve wild animals
Reporting on sex	Female mice were used in the study.
Field-collected samples	The study did not involve field collected samples.
Ethics oversight	The experiments related to mice model was approved by Institutional ethical review committee at University of Health Sciences, Lahore

Note that full information on the approval of the study protocol must also be provided in the manuscript.
